# Physiological and proteomic analyses of the drought stress response in *Amygdalus Mira (Koehne) Yü et Lu* roots

**DOI:** 10.1186/s12870-017-1000-z

**Published:** 2017-02-27

**Authors:** Yuan Cao, Qiuxiang Luo, Yan Tian, Fanjuan Meng

**Affiliations:** 1 College of Life Science, Northeast Forestry University, Harbin, 150040 China; 2 Key Laboratory of Saline-Alkaline Vegetation Ecology Restoration in Oil Field (SAVER), Ministry of Education, Alkali Soil Natural Environmental Science Center, Northeast Forestry University, Harbin, 150040 People’s Republic of China

**Keywords:** *Amygdalus mira (Koehne) Yü et Lu*, Wild peach, Drought, Physiological, Proteomics

## Abstract

**Background:**

Plants are oftentimes exposed to many types of abiotic stresses. Drought is one of the main environmental stresses which limits plant growth, distribution and crop yield worldwide. *Amygdalus mira (Koehne) Yü et Lu* is an important wild peach, and it is considered an ideal wild peach germplasm for improving cultivated peach plants. Because of the loss of genetic variation, cultivated peach plants are sensitive to biotic and abiotic stresses. Wild peach germplasm can offer many useful genes for peach improvement. Responses to drought by withholding water have been studied in *Amygdalus mira (Koehne) Yü et Lu* roots. In this study, plants were divided into well-watered (control) and water-stressed (treatment) groups, and the treatment group did not receive water until the recovery period (day 16).

**Results:**

Several physiological parameters, including root water content and root length, were reduced by drought stress and recovered after rewatering. In addition, the relative conductivity, the levels of proline, MDA and H_2_O_2_, and the activities of ROS scavenging enzymes (POD, APX and CAT) were increased, and none of these factors, except the level of proline, recovered after rewatering. In total, 95 differentially expressed proteins were revealed after drought. The identified proteins refer to a extensive range of biological processes, molecular functions and cellular components, including cytoskeleton dynamics (3.16% of the total 95 proteins), carbohydrate and nitrogen metabolism (6.33% of the total 95 proteins), energy metabolism (7.37% of the total 95 proteins), transcription and translation (18.95% of the total 95 proteins), transport (4.21% of the total 95 proteins), inducers (3.16% of the total 95 proteins), stress and defense (26.31% of the total 95 proteins), molecular chaperones (9.47% of the total 95 proteins), protein degradation (3.16% of the total 95 proteins), signal transduction (7.37% of the total 95 proteins), other materials metabolism (5.26% of the total 95 proteins) and unknown functions (5.26% of the total 95 proteins). Proteins related to defense, stress, transcription and translation play an important role in drought response. In addition, we also examined the correlation between protein and transcript levels.

**Conclusions:**

The interaction between enzymatic and non-enzymatic antioxidants, the levels of proline, MDA, H_2_O_2_ and the relative conductivity, and the expression level of proteins in drought-treated plants all contribute to drought resistance in *Amygdalus mira (Koehne) Yü et Lu.*

**Electronic supplementary material:**

The online version of this article (doi:10.1186/s12870-017-1000-z) contains supplementary material, which is available to authorized users.

## Background

Plants are constantly exposed to many kinds of abiotic stresses [1]. Water deficiency is one of the main environmental stresses which limits plant growth, distribution and crop yield worldwide [2, 3]. It is estimated that, by the end of the 21st century, the droughty terrestrial areas will redouble [4]. Therefore, it is extremely urgent to determine the mechanisms by which plants respond to drought, to improve the tolerance of drought stress.

To deal with water-deficit stress, plants have developed many mechanisms to regulate the balance of cells. Plants optimize their morphology, physiology and metabolism to survive drought stress at both the cellular and organ level [5]. Previous studies have noted that drought stress can induce oxidative stress [6, 7]. Moreover, the glycolate oxidase pathway, which produces H_2_O_2_, is activated by drought [8]. Also, hydroxyl radicals can form from superoxide radicals and H_2_O_2_ which can damage DNA, lipids and proteins [9, 10]. The accumulation of reactive oxygen species (ROS) is frequently caused in cells by drought [4]. An excess of ROS production can lead to oxidative stress in plants and negatively impact the normal function of cells [11]. ROS scavenging ability and subsequent injury-reducing effects may correlate with the tolerance to drought [12]. Both enzymatic and non-enzymatic defense systems have evolved in plants for scavenging and detoxifying ROS. The main non-enzymatic antioxidants in plants are soluble ascorbate and glutathione [13]. ROS scavenging enzymes such as ascorbate peroxidase (APX), superoxide dismutase (SOD), catalase (CAT) and peroxidase (POD) also play a very important role. In enzymatic systems, APX, CAT and POD decompose H_2_O_2_ to H_2_O at different cellular locations [14]. SOD converts O^2−^ to H_2_O_2_ [15]. The balance between antioxidative enzyme activities and ROS production determines the extent of oxidative signaling and/or damage [16]. In addition, as chaperones, the production of HSPs confer plants resistance to stress, can be induced by almost all stresses [17]. Moreover, proline (Pro) plays a very important protective role during drought too. Together with the increase in other osmolytes concentration and proline accumulates will resulted in the decrease of osmotic potential [18]. When plants are under drought stress, this osmotic regulation mechanism makes sure the adaptation to environment [19]. Proline also takes part in the detoxification of ROS [20].

Although there are some researches in woody plants responses to drought in morphological and physiological [21, 22], studies in molecular level is few. It is possible to perform reproducible, quantitative and large-scale research on the effect of every type of stress factor on proteome due to the recent advances in proteomics. Currently, although proteomics has been studied in varieties of plants [23–25], the published proteomic researches on responses to drought followed by recovery is poor [26, 27].

In the atmosphere-plant-soil continuum, the largest hydraulic resistance to water flow is constituted by plant roots. In addition, roots can supply water and nutrients for shoots [28]. The main water-absorbing organs in plants are Roots, and roots play a crucial role in the development of different plant organs because of the direct contact with drying soil [29], and roots are the plant organ most seriously affected by drought. Thus, a variety of stress defense mechanisms against Water deficiency have developed in the root system. Previous researches have demonstrated that drought can induce the stress defense mechanisms in roots, and also the structural adaptation of root architecture too [30–33]. Plants maintain the water uptake through the high hydraulic conductivity, increased rooting depth and root density and osmotic adjustment of the roots [34]. Some studies showed that drought stimulates root growth [35], especially in the deeper soil layers [36]. This mechanism may play an important role in drought resistance [37]. In contrast, some studies found that drought result in restrictions in root growth [38, 39]. However, because of the complexity of phenomena encompassing multiple biochemical and physiological processes at both a cellular and organ level, mechanisms of the biochemical and molecular on drought resistance in plant roots has remained limited so far.

*Amygdalus mira (Koehne) Yü et Lu* is an important wild peach, and it is considered an ideal wild peach germplasm for improving cultivated peach plants. Because of the loss of genetic variation, cultivated peach are sensitive to biotic and abiotic stresses. The wild peach germplasm can offer many useful genes for peach improvement. Our earlier studies have shown that *Amygdalus mira (Koehne) Yü et Lu* in Tibet showed tolerance to drought [40]. In addition, the levels of enzyme activities involved in defense mechanisms markedly increased during drought [40]. However, there is few knowledge available regarding the molecular response mechanisms related to the tolerance of drought in *Amygdalus mira(Koehne) Yü et Lu*. Proteomics approaches are very useful to characterize the responses of plants exposed to water deficiency. Accordingly, we used physiological and proteomic techniques to examine the response of *Amygdalus mira (Koehne) Yü et Lu* to drought conditions. In addition, we also analyzed the capacity of *Amygdalus mira (Koehne) Yü et Lu* to recover following drought. These results will be useful for understanding the mechanisms of drought tolerance in *Amygdalus mira (Koehne) Yü et Lu* and will provide an effective pathway for the exploration of tolerance mechanisms that might improve drought tolerance in peach.

## Methods

### Plant materials and experimental conditions

The experiments were carried out at the Harbin Experimental Forest Farm Greenhouse of Northeast Forestry University in June 2015. The experiments were performed using 60 homogenous plants (1-year-old from seeds). The seeds were obtained from College of Agriculture and Animal Husbandry, Tibet University. The plants were planted in plastic pots (9 cm in bottom diameter, 13.5 cm in upper diameter and 11.5 cm in depth) filled with a 1:3 (v/v) mixture of sand and soil. Potted plants were grown in the greenhouse (day/night air temperature, 28/22 °C; photoperiod, 12 h; 250 μmol photons m^−2^ s^−1^ light; and relative humidity, 60–70%). Plants were divided into two groups: well-watered plants were irrigated every 4 days (control), and water-stressed plants did not receive water until the recovery period (day 16) (treatment).

At each time point (day 4, 8, 12, 16 and 20), the roots of control and treatment plants were harvested. To protect the roots from injury, the soil adhered to the roots was quickly removed by soaking in water, and the roots were then immediately frozen in liquid nitrogen and stored at −80 °C until analysis. Each treatment group was conducted with three independent biological replicates.

### Analysis of physiological parameters

#### Soil water content, root water content and root length

Root length, soil and root fresh weight were measured immediately after sampling. Roots were then dried in an oven at 70 °C for 24 h [41]. Soil and root water content were calculated as follows: Soil water content (%) = (soil fresh weight – dried soil weight)/(soil fresh weight) × 100 (%); and Root water content (%) = (root fresh weight – dried root weight)/(root fresh weight) × 100 (%).

### Measurements of proline, malonaldehyde (MDA), hydrogen peroxide (H_2_O_2_) and relative conductivity

Proline levels were determined using the method of Irigoyen [42]. The roots (0.3 g) were ground to a fine powder in liquid nitrogen and then homogenized in 4 ml ice-cold sulfosalicylic acid (3%, w/v). The homogenate was centrifuged at 12 000 rpm for 10 min at 4 °C and then boiled at 100 °C for 10 min. After cooling, 1 ml of the supernatant was mixed with 1 ml glacial acetic acid and 1.5 ml ninhydrin solution (2.5%, w/v) and then boiled at 100 °C for 30 min. The mixture was then cooled to room temperature, and 3 ml methylbenzene was added. After one hour, the absorbance was read at 520 nm in a UV-1800 spectrophotometer.

MDA content was estimated by the method of Wang [43] with some modifications. The extract was dissolved in 5 ml 10% TCA and centrifuged at 12 000 rpm for 10 min, and the supernatant was then transferred to a 5 ml centrifuge tube and diluted to 4 ml with 10% TCA. The supernatant (1 ml) was mixed with 4 ml 20% TCA containing 0.5% (w/v) thiobarbituric acid (TBA). The mixture was heated in boiling water for 15 min and immediately cooled on ice to stop the reaction; the mixture was then centrifuged at 12 000 rpm for 10 min. The absorbance of the final supernatant was measured at 532 nm, 600 nm and 450 nm. The MDA concentration was calculated by means of an extinction coefficient (155 mM^−1^ cm^−1^).

Hydrogen peroxide (H_2_O_2_) was detected by the method of Sergiev et al. [44]. The finely ground root powder (0.3 g) was homogenized in 0.1% 4 ml trichloroacetic acid (TCA) in an ice bath. After centrifugation at 12 000 rpm for 10 min at 4 °C, 0.5 ml of the supernatant was mixed with 0.5 ml potassium phosphate buffer (50 mM, pH = 6.8) and 1 ml 1 M potassium iodide. After a 5 min reaction, the H_2_O_2_ concentration was calculated based on to a standard curve at 560 nm.

The relative conductivity (REC, %) was assayed following the method of Cavalcanti with some modifications [45]. Roots were cut into pieces and placed in 15 ml deionized water. Then, the mixture was incubated for 5 h at room temperature with shaking. The initial conductivity (*C*_*i*_) was measured using a conductivity meter (Leici-DDS-307). Samples were then boiled at 100 °C for 30 min to completely induce the electrolytes in the solution. After cooling, the conductivity of the killed tissues (*C*_*max*_) was assayed. The relative conductivity (REC, %) was calculated as (*C*_*i*_/*C*_*max*_) × 100 (%).

### Measurement of antioxidant enzyme activities

To measure antioxidant enzyme activities, roots (0.3 g) were ground to a fine powder in liquid nitrogen and dissolved in 2 ml potassium phosphate buffer (50 mM, pH = 7.8).

The activity of superoxide dismutase (SOD) was assayed using the method of Beauchamp and Fridovich (1971) [46]. The assay mixture contained 2.4 ml potassium phosphate buffer (50 mM, pH = 7.8), 0.2 ml 195 mM methionine, 0.2 ml 0.3 mM ethylene diamine tetraacetic acid, 0.2 ml 1.125 mM NBT, 70 μl extraction enzyme and 300 μl 60 μM riboflavin. Enzyme activity was detected at 560 nm by a spectrophotometer.

Ascorbate peroxidase (APX) activity was assayed as previous reported with some modifications [47]. The reaction was started by adding 50 μl extraction enzyme, 1.25 ml potassium phosphate buffer (50 mM, pH = 7.8), 500 μl 2 mM H_2_O_2_ and 200 μl ascorbic acid (ASA), and the decreasing absorbance at 290 nm was monitored for 3 min.

The activity of catalase (CAT) was assayed according to the method of Havir and Mchale with some modifications [48]. The reaction was started by adding 40 μl extraction enzyme, 810 μl 50 mM potassium phosphate buffer (50 mM, pH = 7.8), 500 μl water and 1.5 ml 10 mM H_2_O_2_, and the decreasing absorbance at 240 nm was monitored for 3 min.

The activity of peroxidase (POD) was assayed in 2 ml of potassium phosphate buffer (50 mM) containing 25 μl extraction enzyme, 14 μl guaiacol and 19 μl H_2_O_2_ (30%, v/v) [49]. POD activity was measured at 470 nm.

### Protein extraction

All procedures were performed at 4 °C. Roots (3 g) were ground to a fine powder in liquid nitrogen with a mortar and pestle, and suspended in 15 ml 10% trichloroacetic acid (TCA) containing 0.07% *β*-mercaptoethanol. After vigorous shaking, samples were incubated at −20 °C overnight and then centrifuged for 15 min at 13 500 rpm at 4 °C. The supernatant was discarded. The precipitate was washed three times with cold acetone at −20 °C until the samples became white. The pellet was then freeze-dried and stored at −80 °C. The protein powder was solubilized in lysis buffer (7 M urea, 2 M thiourea, 4% (w/v) CHAPS, 40 mM DTT and 2% (v/v) pH 4–7 IPG buffer) at 37 °C for 1 h, and the insoluble tissue was removed by centrifugation at 13 500 rpm and 4 °C for 30 min. After centrifugation, the protein concentration of the supernatant was determined by the Bradford method with bovine serum albumin (BSA) as a standard [50].

### Two-dimensional electrophoresis (2-DE)

2-DE was carried out according to the method of Wang et al. [43]. The Electrophoreses Power Supply EPS 601 (Amersham Biosciences), Hoefer™ SE 600 Ruby™ electrophoresis unit (Amersham Biosciences) and IPG strips (pH 4–7, 13 cm, GE Healthcare) were used. A mixture of 1000 μg protein sample in 250 μl of a solution containing 7 M urea, 2 M thiourea, 2% (w/v) CHAPS, 40 mM DTT, 0.002% (w/v) bromophenol blue and 0.5% (v/v) IPG buffer, pH 4–7 (GE Healthcare Bio-Sciences Corp., Piscataway, NJ, USA) was prepared. The mixture was loaded onto IPG strips (13 cm, linear pH 4–7, GE Healthcare Bio-Sciences, Uppsala, Sweden). After overnight rehydration of the IPG strips at 20 °C, isoelectric focusing was performed on an Ettan™ IPGphor II™ system (Amersham Biosciences). Focusing was carried out at 20 °C with the following procedure: 100 V for 1 h followed by 500 V for 1 h, 1.5 h linear gradient from 1000 V to 8000 V, and a final 8000 V rapid focus for 5 h. After focusing, the strips were equilibrated in reducing buffer [6 M urea, 50 mM Tris–HCl, pH = 8.8, 2% (w/v) SDS, 30% (v/v) glycerol, 0.002% (w/v) bromophenol blue and 65 mM DTT]. After 15 min, the strips were subsequently equilibrated in alkylation buffer [6 M urea, 50 mM Tris–HCl, pH = 8.8, 2% (w/v) SDS, 30% (v/v) glycerol, 0.002% (w/v) bromophenol blue and 135 mM iodoacetamide]. After 15 min, the equilibrated strips were analyzed by 12.5% sodium dodecyl sulfate-polyacrylamide gel electrophoresis (SDS-PAGE). Gels were stained with Coomassie Brilliant Blue R-250 and destained the next day.

### Gel image analysis

Stained gels were scanned using an image scanner (GE Healthcare, Bio-Sciences, Uppsala, Sweden). Images were analyzed with ImageMaster and Melanie analysis software (Amersham Biosciences, Piscataway, NJ, USA, 2011), including spot detection, background subtraction, volumetric quantification, and matching. Protein spots were selected based on a fold change of ≥ 2 or ≤ 0.5. A threshold of p ≤ 0.05 was used to select differentially expressed protein spots.

### MS analysis and protein identification

The protein spots were manually excised from gels. The gel spots were washed twice, the water was removed, and the gel spots were destained for 5 min at room temperature. Then, the destain solution was removed, and the gel spots were washed twice and incubated in 50% ACN for 5 min. The 50% ACN was then removed and replaced with 100% ACN for 5 min. The gels were rehydrated in 2–4 μl trypsin (Promega, Madison, USA) solution (20 μg/ml in 25 mmol/l NH_4_HCO_3_) for 30 min. Next, 20 μl cover solution (25 mmol/l NH_4_HCO_3_) was added, and the gels were digested for 16 h at 37 °C. The supernatants were transferred to a new tube, and the gels were extracted once with 50 μl extraction buffer (67% ACN and 5% TFA). The peptide extracts and the supernatants of the gel spots were combined and then completely dried.

Samples were re-suspended with 5 l 0.1% TFA followed by mixing in 1:1 ratio with a matrix consisting of a saturated solution of α-cyano-4-hydroxy-trans-cinnamic acid in 50% ACN and 0.1% TFA. One microliter of the mixture was spotted on a stainless-steel sample target plate. Peptide MS and MS/MS were performed on an ABI 4800 MALDI-TOF/TOF Plus mass spectrometer (Applied Biosystems, Foster City, USA). Data were acquired in a positive MS reflector using a CalMix5 standard to calibrate the instrument (ABI4800 Calibration Mixture). Both the MS and MS/MS data were integrated and processed by using the GPS Explorer V3.6 software (Applied Biosystems, USA) with default parameters. Based on combined MS and MS/MS spectra, proteins were successfully identified using a 95% or higher confidence interval of their scores in the Mascot V2.3 search engine (Matrix Science Ltd., London, U.K.) with the following search parameters: NCBI non-redundant database; trypsin as the digestion enzyme; one missed cleavage site; partial modifications of Carbamidomethyl (C) and Oxidation (M); 60 ppm for precursor ion tolerance; and 0.25 Da for fragment ion tolerance.

### Quantitative real-time PCR (RT-PCR)

To investigate the relationship between the transcriptional and translational expression of related genes after treatment, we used qRT-PCR to analyze 11 genes selected based on the proteomics results (Additional file 1: Table S1). Total RNA was isolated using a plant RNA extraction kit (Biotecke, China), and cDNA was synthesized from 1 μg of the total RNA with PrimeScript Reverse Transcriptase (Takara, Japan) according to the manufacturer′s instructions. Specific primer pairs for the selected genes were designed by comparing the nucleotide sequences of the conserved region of different species, such as *Prunus Linn*, *Amygdalus Linn*, *Pyrus Linn* and *Malus Mill,* of the Rosaceae family, to which *Amygdalus mira (Koehne) Yü et Lu* belongs, using BioEdit and Primer Premier 5.0 software (Additional file 2: Table S2). The qRT-PCR was performed using SYBR Green Real-time PCR Master Mix (Toyobo, Japan) with a LightCycler480 (Roche, USA), with semi-quantitative PCR first used to test the primer pairs and confirm the annealing temperatures (Additional file 2: Table S2). The expression level of the ACTIN gene was used as an internal control (reference gene). Relative expression of the target genes was calculated using the comparative Ct method.

### Statistical analysis and experimental design

The experiment had two treatments (control and drought) at five time points (4, 8, 12, 16 and 20 days) with three independent replicates for each condition. Data were analyzed using analysis of variance (ANOVA), and the means were compared using Tukey’s test (*p* < 0.05).

## Results

### Morphological responses to drought stress and recovery

**Fig. 1:**
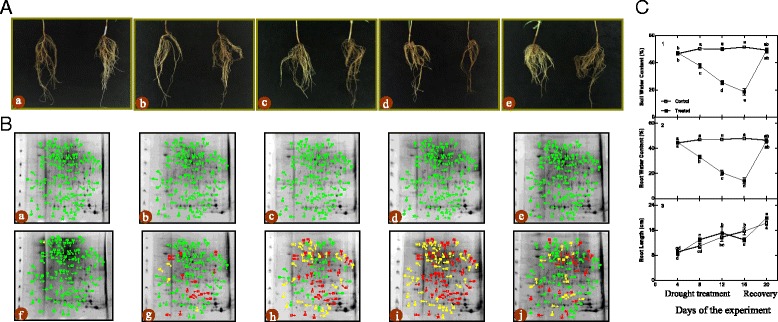
Changes of morphological (**a**), 2-DE master gel of roots (**b**), soil water content, root water content and root length (**c**) of *Amygdalus mira (Koehne) Yü et Lu* roots during drought stress and recovery period

To confirm how drought stress and recovery influence the roots of *Amygdalus mira (Koehne) Yü et Lu*, firstly, we surveyed the morphological responses at 5 time points (day 4, 8, 12, and 16 after watering and at day 20, after rewatering on day 16). As shown in Fig. 1, panel a, the roots became shriveled and brown during drought stress, especially at day 16. After rewatering, on day 20 the roots appeared to recover from the drought stress. Soil water content, root water content and root length were consistent with the morphological response (Fig. 1, panel c). Root water content and root length were decreased by approximately 70.44% and 17.47%, respectively, in drought-stressed plants compared with the control plants at day 16. In addition, root water content and root length in drought-stressed plants recovered after rewatering.

### Physiological and biochemical responses to drought stress and recovery

**Fig. 2:**
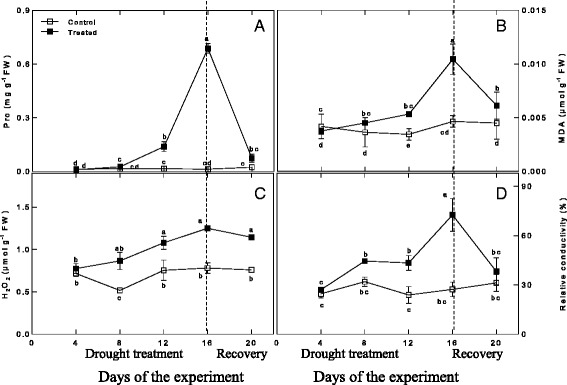
Changes in Pro (**a**), MDA (**b**), H_2_O_2_ (**c**), and relative conductivity (**d**) during drought treatment and recovery

Regarding physiological and biochemical responses, *Amygdalus mira (Koehne) Yü et Lu* roots showed accumulation of proline, MDA and H_2_O_2_ after drought stress, with the levels of proline, MDA and H_2_O_2_ increasing 52.38-, 2.25- and 1.60-fold, respectively, in drought-stressed plants compared with the control group at day 16 (Fig. 2, panel a, b, c). After rewatering, the proline level returned to normal, but the levels of H_2_O_2_ and MDA were still higher than those of the control group (Fig. 2, panel a, b, c). The variations in electrolyte leakage were similar to the variations observed in proline levels (Fig. 2, panel d).

**Fig. 3:**
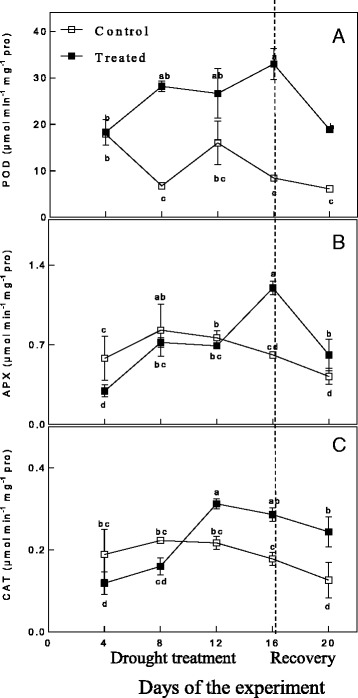
Changes in POD (**a**), APX (**b**), and CAT (**c**) during drought treatment and recovery

The activities of ROS scavenging enzymes (POD, CAT and APX) in response to drought stress and recovery are depicted in Fig. 3. The activity of POD under drought stress was higher at all time points compared to the control group and increased with the duration of water stress, reaching the highest level at day 16 (approximately 3.91-fold higher than the control level). After rewatering, POD activity decreased to the initial level but was still higher than that of the control group (approximately 3.10-fold higher than normal). The activities of CAT and APX under drought conditions were initially lower than those of the control groups. The CAT activity reached a maximum level in the drought-stressed plants at day 12, at which point it was higher than the level in the control group, while the APX activity on day 12 in drought-stressed plants was still lower than that of the control group. From day 12 to day 16, the CAT activity decreased, while in contrast, the APX activity significantly increased. After rewatering, the activity of CAT did not recover, while the activity of APX decreased significantly, though it was still higher than that of the control group.

### Identification of differentially expressed proteins in response to drought stress and recovery

Based on the biochemical, physiological and morphological responses to drought stress and recovery, we chose two time points, 16 days, which was the longest drought duration, and 20 days, which was during the recovery period after rewatering, to profile the changes of drought-responsive and recovery-responsive proteome. In total, 95 significantly differently expressed proteins were revealed in the drought stress groups compared to control groups (Fig. 1, panel b and Additional file 1: Table S1). On day 16, 47 of the 95 identified proteins (49.47%) were up-regulated while the rest were down-regulated in drought-stressed plants (S16) relative to their control group (C16). After rewatering, in the drought-stressed plants compared with the control group, we found 22 down-regulated proteins (22.92%), 18 up-regulated proteins (18.75%) and 56 proteins (58.33%) that were not significantly differently expressed in the S20 group. Comparing the expression of these proteins on day 16 and day 20, 16 proteins (16.84%) were up-regulated in the S16 group and down-regulated in the S20 plants, 9 proteins (9.47%) were down-regulated in S16 and up-regulated in S20, 9 proteins (9.47%) were up-regulated in both S16 and S20, 6 proteins (6.32%) were down-regulated in both S16 and S20, 22 proteins (23.16%) were up-regulated in S16 but not significantly differentially expressed in S20 and 33 proteins (34.74%) were down-regulated in S16 but not significantly differentially expressed in S20.

**Fig. 4:**
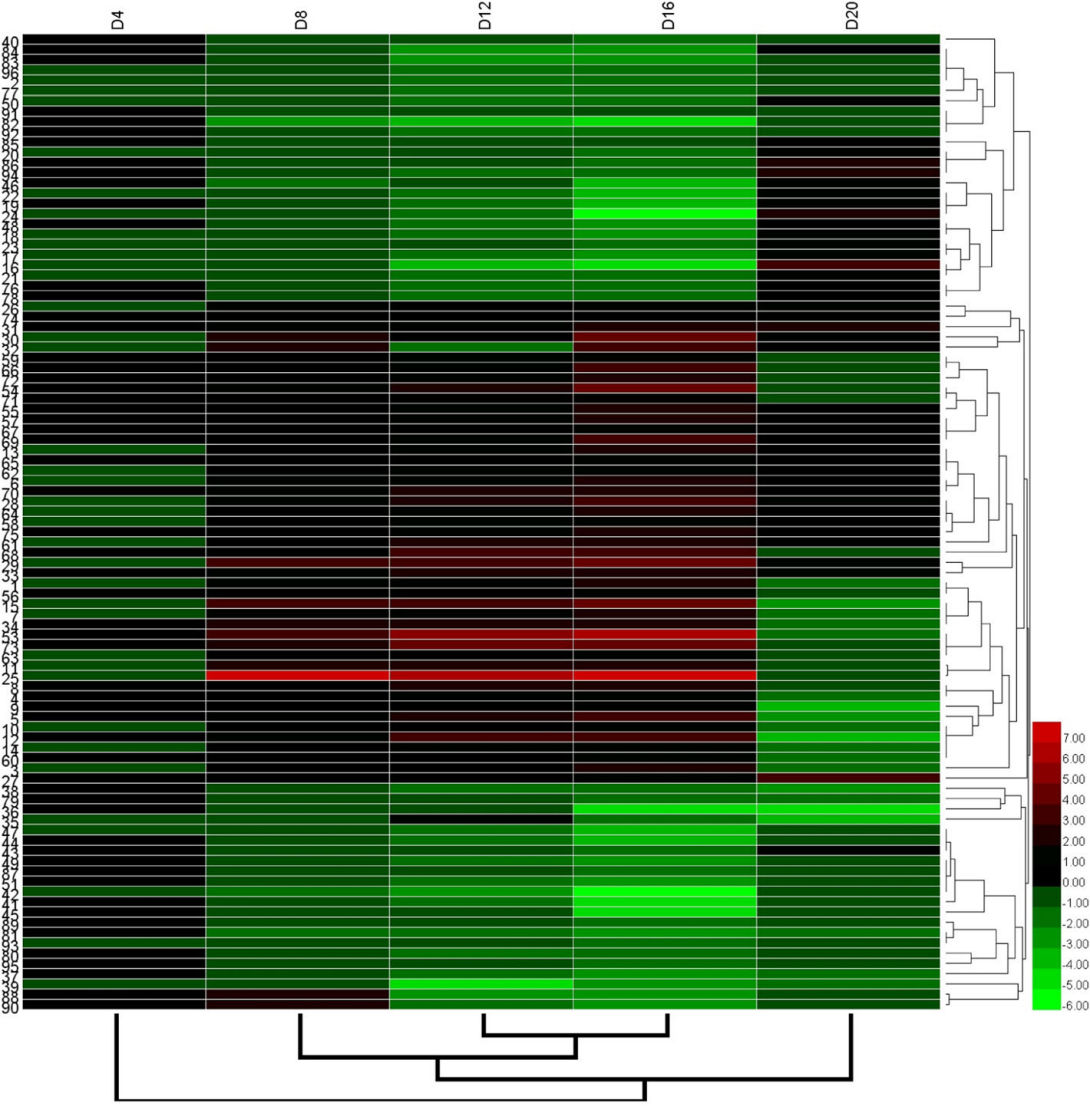
Hierarchical clustering of the 95 differentially expressed proteins

We also assessed how protein level changes during water deficiency and recovery differed at the five time points. As stress progressed and through recovery period, from the day 4 to day 20, there were differences (decreasing or increasing) between control groups and stress groups in protein levels (Additional file 3: Table S3). In addition, we followed up our analysis of [log_2_ (fold protein level changes)] in the process of drought and recovery by using κ-means clustering (Fig. 4).

**Fig. 5:**
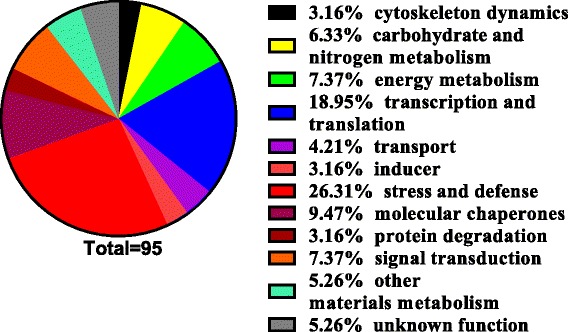
Functional category distribution of the 95 identified proteins

Because the genome and proteome of *Amygdalus mira (Koehne) Yü et Lu* have not been widely characterized, and the number of protein entries in public databases is quite low, it can be tolerated that use the primer sequence and protein entries for similar oligonucleotide primers and proteins expressed in Rosaceae family species such as *Prunus persica*, which is bound up with *Amygdalus mira (Koehne) Yü et Lu*, provided that a reasonable abundant sequence homology by amino acid replace or deletion [51]. In our study, we analyzed 95 proteins by MS analysis. The best matched protein with the highest score was selected as the final result for every protein spot (Additional file 1: Table S1). The identified proteins refer to a extensive range of biological processes, molecular functions and cellular components including cytoskeleton dynamics (3.16% of the total 95 proteins), carbohydrate and nitrogen metabolism (6.33% of the total 95 proteins), energy metabolism (7.37% of the total 95 proteins), transcription and translation (18.95% of the total 95 proteins), transport (4.21% of the total 95 proteins), inducer (3.16% of the total 95 proteins), stress and defense (26.31% of the total 95 proteins), molecular chaperones (9.47% of the total 95 proteins), protein degradation (3.16% of the total 95 proteins), signal transduction (7.37% of the total 95 proteins), other materials metabolism (5.26% of the total 95 proteins) and unknown function (5.26% of the total 95 proteins) (Fig. 5).

### Proteins related to cytoskeleton dynamics

Three proteins (Spot. 40, profilin; Spot. 84, actin 1; and Spot. 85, ACT1) related to cytoskeleton dynamics were identified (Additional file 1: Table S1). The expression of all three decreased due to drought stress and recovered after rewatering (Additional file 3: Table S3).

### Proteins related to carbohydrate and nitrogen metabolism

Several proteins related to carbohydrate and nitrogen metabolism were found to be drought-responsive in the roots of *Amygdalus mira (Koehne) Yü et Lu* (Additional file 1: Table S1). The levels of four proteins (Spot. 20, 38, 91 and 92) were decreased and the levels of two proteins (Spot. 26 and 59) were increased by drought stress. After rewatering, the expression of three of the six proteins (Spot. 59, 91 and 92) returned to their original levels, one protein (Spot. 20) became up-regulated, and the expression of the other two proteins (Spot. 26 and 38) remained the same as before rewatering (Additional file 3: Table S3).

### Proteins related to energy metabolism

Seven proteins related to energy metabolism were identified (Additional file 1: Table S1). The levels of the ATP synthase beta subunit (Spot. 1 and 56) and cytochrome P_450_ (Spot. 13) were increased during drought stress, while the levels of the other four proteins (Spot. 46, 48, 47 and 22) were decreased. After rewatering, two proteins (Spot. 46 and 22) were up-regulated, while five proteins (Spot. 1, 13, 48, 56 and 47) returned to normal levels (Additional file 3: Table S3).

### Proteins related to transcription and translation

Large numbers of transcription- and translation-related proteins were identified during drought stress (Additional file 1: Table S1). Drought stress resulted in an increase in the abundance of twelve proteins (Spot. 3, 8, 9, 28, 29, 54, 57, 58, 61, 64, 66 and 69), while the expression of six other proteins (Spot. 19, 49, 23, 76, 79 and 89) decreased. After rewatering, the expression of three proteins (Spot. 3, 9 and 79) decreased, four (Spot. 19, 28, 29 and 23) increased, and the rest (Spot. 8, 49, 54, 57, 58, 61, 64, 66, 69, 76 and 89) returned to normal levels (Additional file 3: Table S3).

### Proteins related to transport

Four transport-related proteins were identified as differentially expressed due to drought stress (Additional file 1: Table S1). Two of these proteins (Spot. 4 and 5) were increased and the other two (Spot. 17 and 83) were decreased by drought stress. However, after rewatering, two proteins (Spot. 4 and 5) were down-regulated, one protein (Spot. 17) was up-regulated, and one protein (Spot. 83) returned to the level of the control group (Additional file 3: Table S3).

### Proteins related to inducers

There were three spots identified that were related to the inducer category (Additional file 1: Table S1), and all three were induced stolon tip protein PJ-1. However, interestingly, they exhibited different responses to drought stress in our study. Spot. 10 was up-regulated during drought stress and down-regulated at day 20. Meanwhile, Spot. 37 was down-regulated during drought stress and remained down-regulated after rewatering. However, Spot. 35 was increased at day 12 and decreased at day 16 of drought stress and remained down-regulated after rewatering (Additional file 3: Table S3). The reason behind these different responses remains unclear and requires further investigation.

### Proteins related to stress and defense

Large numbers of stress and defense-related proteins were found to be induced in the drought-stressed roots (Additional file 1: Table S1), including five ROS metabolism-related proteins (Spot. 2, 44, 63, 73 and 88). SOD (Spot. 2) and POD (Spot. 44) were both down-regulated by water deficiency and recovered after rewatering. However, APX (Spot. 63) responded differently. APX was up-regulated by drought stress and recovered by rewatering. CAT expression exhibited a trend similar to the proteins related to inducer. Two different spots (Spot. 73 and 88) were both CAT, and responded differently to drought stress. Spot. 73 was up-regulated during drought, but Spot. 88 only exhibited up-regulation at day 8, after which it was down-regulated. Both spots returned to control levels after rewatering. Twelve of the other twenty stress and defense proteins (Spot. 6, 11, 12, 14, 15, 27, 31, 34, 53, 55, 68 and 60) were increased and the remaining eight (Spot. 18, 24, 36, 39, 50, 51, 77 and 86) were decreased by drought stress. After rewatering, there were five proteins (Spot. 18, 24, 27, 31 and 86) that were up-regulated, eight proteins (Spot. 12, 14, 15, 34, 36, 39, 53 and 60) that were down-regulated and seven proteins (Spot. 6, 11, 50, 51, 55, 68 and 77) that returned to normal levels (Additional file 3: Table S3).

### Proteins related to molecular chaperones

Nine molecular chaperones were identified in our study (Additional file 1: Table S1). Three of them (Spot. 30, 32 and 75) were up-regulated by drought stress, and two of these three (Spot. 32 and 75) recovered after rewatering, while the level of Spot. 30 was still higher than the control level after rewatering. The other six proteins (Spot. 78, 80, 81, 82, 94 and 96) showed an opposite response to drought. In addition, after rewatering, four of the six (Spot. 78, 80, 82 and 96) recovered, one protein (Spot. 81) was still lower than the control level and one protein (Spot. 94) was up-regulated (Additional file 3: Table S3).

### Proteins related to protein degradation

Three protein degradation-related proteins were identified as drought-responsive proteins in our study (Additional file 1: Table S1). Predicted: putative DNA repair protein RAD23-3-like (Spot. 16) was significantly down-regulated by drought stress and up-regulated by rewatering. The level of proteasome subunit alpha type-5 (Spot. 72) was increased during drought stress and recovered after rewatering. Remarkably, the abundance of the RAD23 protein (Spot. 25) was significantly increased (approximately 363-fold) by drought stress at day 16 and recovered after rewatering (Additional file 3: Table S3).

### Proteins related to signal transduction

Seven proteins related to signal transduction were found to be differentially expressed in the drought-stressed roots (Additional file 1: Table S1). Three of seven (Spot. 33, 62 and 74) were increased in response to drought, and only one protein (Spot. 33) did not recover after rewatering. The other four proteins (Spot. 21, 41, 43 and 93) were down-regulated due to drought, and only one protein (Spot. 21) did not recover and was higher than the control level after rewatering (Additional file 3: Table S3).

### Proteins related to other materials metabolism

Five other materials metabolism-related proteins were identified as drought-responsive proteins in roots (Additional file 1: Table S1). The levels of enoyl-ACP reductase family proteins (Spot. 87 and 90) were reduced by drought stress and recovered after rewatering. The other three other materials, metabolism-related proteins (Spot. 7, 65 and 70), were up-regulated by drought, and only one protein (Spot. 7) did not recover and was lower than the control level after rewatering (Additional file 3: Table S3).

### Unknown functions

Five proteins with unknown functions were identified as drought-responsive proteins in our study (Additional file 1: Table S1). Three of them (Spot. 42, 45 and 95) were down-regulated by drought, while the other two (Spot. 67 and 71) were up-regulated under drought. Moreover, the levels of all five unknown function proteins recovered after rewatering (Additional file 3: Table S3).

### Comparison of transcription data with protein expression data

**Fig. 6:**
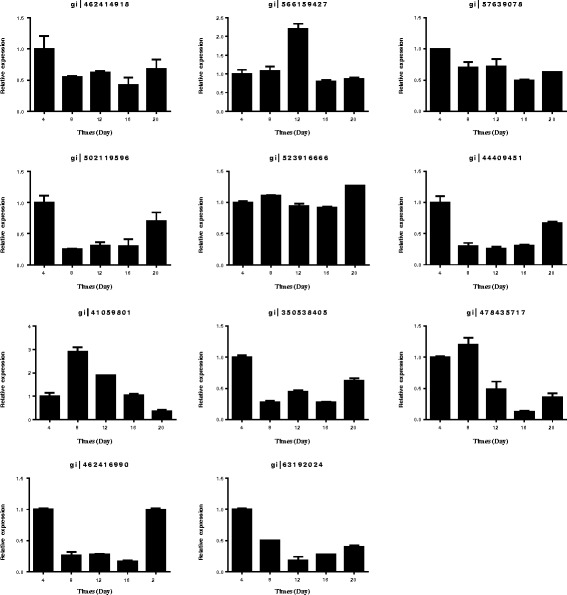
Relative gene expression analyses of 11 differentially expressed proteins by quantitative real-time PCR

To determine whether changes of gene transcription levels correlated with changes of protein levels, a quantitative real-time PCR analysis of 11 genes was performed (Additional file 4: Figure S4 and Fig. 6). Six genes (Spot. 11, 42, 45, 51, 70 and 76) were down-regulated by drought stress and recovered by rewatering at the mRNA level, and four of them (Spot. 42, 45, 51 and 76) showed similar results to the protein analysis. One gene (Spot. 15) showed no significant changes at the mRNA level but did exhibit alterations in protein levels. In addition, two genes (Spot. 90 and 59) were down-regulated at the mRNA level both under drought and after rewatering, which was inconsistent with the protein levels. Spot. 61 exhibited the highest mRNA and protein expression at day 12 of drought, with the mRNA expression, but not the protein expression, recovering at day 16. The mRNA expression of Spot. 75 was up-regulated in response to drought at day 8 but recovered at subsequent drought periods and decreased after rewatering, which was different than the trend observed at the protein level.

## Discussion

Biochemical, physiological and molecular influences on plants are wide-spread during drought stress and can be divided into three aspects: growth control, stress damage control and osmotic homeostasis [52]. An integrated proteomics, biochemical, physiological and morphological approach was used for our research to investigate these three aspects of drought stress responses in *Amygdalus mira (Koehne) Yü et Lu*.

### Morphological, physiological and biochemical responses to drought stress and recovery

As the organs in direct contact with soil, roots are seriously affected by drought stress. Previous studies displayed that root growth was significantly enhanced at the early stage of drought [29, 53], but in our study, there were no significant changes at the early phase of drought. Yoshimura [26] observed that wild watermelon’s root growth suppressed at the later phase of drought, which is consistent with our study. We identified significant suppression of root length at day 16, which Yoshimura [26] hypothesized may be relevant to the implementation of drought tolerance mechanisms as roots deal with the reduced soil water potential without obtaining new water resources. After rewatering, at day 20, root growth recovered. Meanwhile, root water content gradually decreased with intensified drought stress, which is also consistent with the previous study [54]. After rewatering, root water content also recovered.

Proline plays a protective role during drought stress [55]. Nayyar [51] found a higher rate of proline accumulation and utilization during drought in wheat. Similar results have also been obtained in alfalfa [56]. Moreover, decreased membrane injury correlated with a greater ability to accumulate proline has been found in barley [57]. In the current study, drought stress induced a 30-fold increase in proline levels at day 16. Good [18] suggested that proline level increases may be primarily the result of increased synthesis. After rewatering, proline levels decreased and showed no significant difference compared to the control group. MDA is a product of lipid peroxidation, and the degree of membrane lipid peroxidation can be reflected by MDA levels [10]. As a ROS, H_2_O_2_ can damage membrane lipids, proteins and DNA [8, 9, 11]. In other words, the levels of MDA and H_2_O_2_ can play a role of indicators of the free radical reactions occurring in the stressed tissue [58]. Additionally, the relative conductivity (REC) is another indicator of membrane damage [59]. Previous studies reported that the levels of MDA [10, 59, 60], H_2_O_2_ [61, 62] and REC [10, 59] significantly increased in response to drought. As expected, levels of all three indicators increased significantly during drought in our research. In particular, the levels of MDA and H_2_O_2_ were significantly increased after 8 days of drought, while the REC level exhibited significant changes after 12 days of drought. All these changes indicated that drought stress led to membrane damage. After rewatering, the level of REC returned to normal, the level of MDA significantly decreased but was still higher than that of the controls, and the level of H_2_O_2_ did not recover. From these results, we can conclude that the membrane damage of membrane was being repaired but that the repair process was not complete. The signaling in plants or potential of oxidative stress may be indicated by the level of ROS during drought and recovery [63]. Sofo [64] and Upadhyaya [65] found lower ROS levels in *Prunus* hybrids and Tea, respectively. In contrast, Bian [16] found that the accumulation of ROS still appeared and RWC had fully recovered, which is consistent with our result. It may because that it did not necessarily limit production of ROS during the recovery period in *Amygdalus mira (Koehne) Yü et Lu* roots, suggesting that oxidative stress is involved in root recovery from drought. The role of ROS in drought and subsequent recovery of *Amygdalus mira (Koehne) Yü et Lu* roots remains unclear and requires further investigation.

The increased ROS products induced changes in the activities of antioxidant enzymes. Antioxidant enzymes can maintain the balance of the formation and elimination of ROS by detoxification of excess ROS [66]. CAT is present in peroxisomes, but it is essential for resolving H_2_O_2_ during stress [67]. APX is an antioxidant enzyme in the ascorbate-glutathione (ASA-GSH) cycle, which is an efficient antioxidant system for the detoxification of H_2_O_2_ [68]. The ASA-GSH cycle properly scavenges ROS in plant cells by maintaining a ratio of a reduced per oxidized ascorbic acid and glutathione [8]. Several previous studies found that APX activity was increased [64], CAT activity was reduced [10, 59] or increased [69] and POD activity was increased [70] during drought stress, and upon rewatering, POD activity significantly declined but was still higher than that of the control, while APX activity was down-regulated. These different responses may depend on intensity of ROS production, stress severity and plant species [16]. In the present research, the activities of APX, CAT and POD were all up-regulated during drought stress. This result suggested that *Amygdalus mira (Koehne) Yü et Lu* up-regulates the activities of these antioxidant enzymes to protect against ROS toxicity. After rewatering, the activities of POD and APX significantly declined but were still significantly higher than those of the control, and the activity of CAT did not show a significant change. This result has been previously demonstrated [16]. It has been indicated that although there have different affinities for H_2_O_2_ in POD, APX and CAT, they can all efficiently facilitate H_2_O_2_ scavenging in *Amygdalus mira (Koehne) Yü et Lu* root cells. In addition, a co-regulated antioxidant mechanism could develop to vary with roots in *Amygdalus mira (Koehne) Yü et Lu*.

### Differentially expressed proteins in drought stress and recovery

Actin (spot. 84 and 85) is a typical cytoskeleton-related protein, and profilin (spot. 40) plays an important role in the regulation of actin polymerization [71]. Liu [71] found that the down-regulation of profilin lead to the number of filamentous actin decreased and induced actin disorganization. Previous studies reported that profilin significantly accelerates formin-mediated barbed end actin elongation [72, 73]. In our research, the expression of these proteins was down-regulated in response to drought and returned to normal levels after rewatering compared to the controls. This result is consistent with the morphological response. From these observations, we can conclude that drought caused down-regulated expression of proteins related to cytoskeleton dynamics, resulting in shriveled and brown *Amygdalus mira (Koehne) Yü et Lu* roots.

In previous studies, the levels of carbohydrate and nitrogen metabolism-related proteins were increased because of drought stress [74, 75]. On the other hand, in other studies, it has been reported that the expression of these proteins decreased in response to drought stress [76, 77]. In carbohydrate metabolism, Glycolysis is an important metabolic pathway which can be found in almost all living organisms. The central role of glycolysis is to generate precursors for anabolism and provide energy to plants [78]. In our study, the expression of glyceraldehyde-3-phosphate dehydrogenase (Spot. 91), which is involved in glycolysis, was decreased by drought and recovered after rewatering. As a typical glycolytic enzyme, glyceraldehyde-3-phosphate dehydrogenase plays an important role in response to stress and the development of plants [79]. It has been reported that the accumulation of carbohydrate metabolism enzymes can be displayed in the early period of drought, particularly in tolerant species, and then reduced when drought is more invasive or the plant species is less tolerant to drought [27]. In our study, four proteins (Spot. 20, 38, 91 and 92) were down-regulated and two proteins (Spot. 26 and 59) were up-regulated after drought stress, and three of these six proteins (Spot. 20, 26 and 38) did not recover after rewatering. This result suggested that these proteins act synergistically to protect the plant from drought stress, and this effect could mean a better reaction capacity or higher flexibility for Spot. 59, 91 and 92 compared to spot. 20, 26 and 38.

Two identified proteins (Spot. 1 and 56, ATP synthase beta subunit) are related to ATP synthesis, which is applied to carbon assimilation in the light-reactions of PS [80]. ATP synthase is a key enzyme for ATP synthesis during electron transport. With the predominantly on the beta subunit or catalytic sites being carried wholly, the activity and stability of ATP synthase regulate the ATP synthesis [81]. Previous studies regarding the expression of ATP-related proteins in response to drought stress are contradictory. José [82] and Tezara [83] observed a decrease in the expression of the ATP synthase beta subunit during drought stress. They hypothesized that because a smaller amount of energy is needed by the cells during drought in these plants, the ATPase content is likely reduced. However, Kottapalli [84], and Zhou [85] observed the opposite, and these results are consistent with our current study. Kottapalli [84] suggested that the ATP synthase beta subunit is highly induced only in drought-tolerant genotypes. In our study, the expression of this ATP-related protein is up-regulated during drought stress and recovers after rewatering. The higher expression of the ATP synthase beta subunit in our study might improve the energy supply to protect *Amygdalus mira (Koehne) Yü et Lu* from injury under drought stress conditions. Cytochrome P_450_ (Spot. 13) is a protein that catalyzes the transformation of teasterone to 3-dehydroteasterone as well as the transformation of 6-deoxoteasterone to 3-dehydro-6-deoxoteasterone late in the brassinosteroid (BR) biosynthesis pathway [86]. To protect plants from environmental stresses, BR has biological activities that include altering plant metabolism [84]. Farah [10] found that cytochrome P_450_ was up-regulated during drought, and it is consistent with our study. Moreover, Hong [86] found that a cytochrome P_450_ loss-of-function mutant in rice shows reduced BR biosynthesis and a dwarf phenotype. As a mechanism in plants of drought, changes in BR biosynthesis remain to be analyzed.

In the signal transduction network, transcription factors are essential, and they lead from the perception of stress signals to the expression of stress-responsive genes [10]. Our research found a significant increase in ethylene-responsive transcription factor 1A-like (Spot. 28) during drought, and it is consistent with the Farah study [10]. In addition, the expression level did not recover to normal compared with the control after rewatering. Ethylene-responsive transcription factor 1A-like (ERTF) is involved in a variety of plant reactions to abiotic or biotic stresses, and Seo [87] found that over-expression of the ERTF gene led to tolerance improvement to drought stress. It has been reported that the levels of some proteins related to transcription and translation are up-regulated by stress to enhance stress resistance during a lot of defense-related proteins are newly produced [25]. In our study, ribosomal protein S18 (Spot. 54) and elongation factor Tu family protein (Spot. 61) increased under drought stress, with the expression of ribosomal protein S18 increasing to almost 32 times the control level at day 16 of drought treatment. This result suggested that to enhance drought stress resistance, *Amygdalus mira (Koehne) Yü et Lu* produced defensive proteins through the up-regulation of these transcription- and translation-related proteins.

It is well known that the enhanced production of ROS accompanies drought [26]. The first step of enzymatic antioxidant defense response is the conversion of superoxide to hydrogen peroxide by superoxide dismutase [Cu-Zn] 1 (Cu-Zn SOD, Spot. 2) [88]. Zhou [85] indicated that a major SOD isoform contributing to sustained SOD activity is Fe SOD, but previous studies found that Cu-Zn SOD (Spot. 2) primarily responds to drought [89, 90]. Brossa [91] found that SOD was up-regulated during drought; however, in our study, consistent with what Zhou [85] found, the expression of SOD exhibited a trend toward recovery after rewatering. Ascorbate peroxidase (APX, Spot. 63) plays a very important role in removing H_2_O_2_ by utilizing ASA to reduce H_2_O_2_ to H_2_O. The responses of catalase isozyme 2 (CAT 2, Spot. 73 and 88) to drought are heterogeneous, and it has been shown to remain unchanged, increase or even decrease under drought stress [92]. The regulation of CAT under drought is complex [91]. Peroxisomal membrane protein PMP22 (Spot. 44) is a component of peroxisomes, (pod) which contain antioxidant enzymes. Yoshimura [26] found POD was up-regulated both at the early phases and the late phases of drought stress in wild watermelon. In our study, during drought, the expression of APX (Spot. 63) was up-regulated, and the expression of peroxisomal membrane protein PMP22 (Spot. 44) significantly decreased. After rewatering, the expression of APX (Spot. 63) was lower than that of the controls while the expression of peroxisomal membrane protein PMP22 (Spot. 44) returned to the level of the controls. The expression of CAT 2 (Spot. 73 and 88) in our study is interesting; Spot. 73 showed a significant increase, while Spot. 88 was decreased at the late stage of drought stress, and both were lower than the control levels after rewatering. Zhou [85] found a similar result in APX protein expression. This result implied that CAT protein in *Amygdalus mira (Koehne) Yü et Lu* was distributed in different cell compartments and had different tasks under drought stress [93]. In contrast to the activities of POD, APX and CAT, changes in the expression levels of these proteins were not the same in response to drought. It revealed that protein levels do not necessarily correlate with protein activities. Due to the drought acclimation phases, developmental phases and species research, there are certainly variations in response to drought [94]. The induction of ROS-related factors suggests that the production of ROS is accompanied with drought, and the induction of ROS-related enzymes indicates an antioxidant system that may be involved in the protection of *Amygdalus mira (Koehne) Yü et Lu* from damage due to drought stress.

Molecular chaperones were also identified to be regulated by drought stress. Previous studies found that molecular chaperones were up-regulated during drought stress [1, 26, 91]. In our study, three types of molecular chaperones (Spot. 30, 32 and 75) were found to be up-regulated, and the other six identified chaperones (Spot. 78, 80, 81, 82, 94 and 96) were shown to be down-regulated. Only luminal-binding protein 5 (Spot. 96) recovered after rewatering. Among the identified chaperones, HSP70 (Spot. 78, 81 and 82) has been shown to refold non-native proteins which facilitate translocation processes and prevent protein aggregation under stress [95]. Furthermore, small HSPs (Spot. 32 and 75) have been known to through protecting NADH:ubiquinone oxidoreductase activity (Complex I) to maintaining electron transport in mitochondrial during stress [96]. Our results suggest the important roles of this family in coping with drought by the regulation of molecular chaperones. In addition, our data indicated that cellular proteins in *Amygdalus mira (Koehne) Yü et Lu* roots are potentially exposed to an increasing risk of aggregation and denaturation during gradual drought stress, and the induction of molecular chaperones may have an essential role in offsetting this risk.

The observed expression pattern during drought stress for the proteins involved in protein degradation was also complex. RAD23 protein (Spot. 25) and predicted: putative DNA repair protein RAD23-3-like (Spot. 16) have been known to function in DNA excision repair, and both contain a ubiquitin-like domain. As Hershko [97] indicated, protein degradation via the ubiquitin-proteasome pathway plays a key role in controlling cellular processes in eukaryotic cells. Proteasome subunit alpha type-5 (Spot. 72) has been reported to increase under stress [23, 26]. In our study, we observed significant up-regulation and down-regulation of Spot. 25 and Spot. 16, respectively. Interestingly, after rewatering, the expressions of these two proteins were different than the expressions under drought stress. After rewatering, RAD23 protein (Spot. 25) was decreased and predicted: putative DNA repair protein RAD23-3-like (Spot. 16) was increased. It is possible that proteins related to protein degradation are also connection with the biosynthesis of novel proteins contained in the drought resistance mechanisms in *Amygdalus mira (Koehne) Yü et Lu* roots.

### Quantitative real-time PCR analysis

The transcription level of seven genes was different from the protein level, an inconsistency that has been observed in many previous studies [98, 99]. The differences may be due to post-translational processing or post-transcriptional regulation [100]. The consistency between protein expression level and transcription level in the other four analyzed genes manifests that these proteins may be initially accommodated at the transcriptional level during root development phase [98].

## Conclusions

Our research supports further information about proteomic, biochemical, physiological and morphological responses in the roots of *Amygdalus mira (Koehne) Yü et Lu* to drought and recovery. At the physiological level, drought stress reduced root water content and root length, and *Amygdalus mira (Koehne) Yü et Lu* responded to drought by increasing the levels of proline, MDA, H_2_O_2_ and the relative conductivity. The activity of POD, APX, and CAT in roots increased when exposed to drought and did not recover after rewatering. By analyzing proteins in the treatment and control groups over time, we support quantitative evidence regarding how biological processes are regulated during gradual drought and rewatering. Moreover, if this is a single time point experiment, such information would be missed. Plenty of proteins have been identified to be contained in drought stress. In addition, we also presented a correlation between protein and transcript levels. Generally, the interaction between enzymatic and non-enzymatic antioxidants, the levels of proline, MDA, H_2_O_2_ and the relative conductivity, and the expression level of proteins in drought-treated plants all contribute to drought resistance in *Amygdalus mira (Koehne) Yü et Lu*. However, a more comprehensive analysis is necessary for understanding the variability in the response of *Amygdalus mira (Koehne) Yü et Lu* to drought.

## Additional files

Additional file 1: Table S1. Identification of drought-responsive proteins in *Amygdalus mira (Koehne) Yü et Lu* roots by mass spectrometry (MS) analysis. (DOC 2010 kb)
Additional file 2: Table S2. The primer sequences for real time PCR. (DOC 41 kb)
Additional file 3: Table S3. Proteins differentially expressed in *Amygdalus mira (Koehne) Yü et Lu* during drought and recovery. (DOC 229 kb)
Additional file 4: Figure S4. Semi-quantitative PCR analysis of eleven genes and *ACTIN* of *Amygdalus mira (Koehne) Yü et Lu* roots during drought stress and recovery period, respectively. (DOC 201 kb)

## Abbreviations

APX: Abscisic acid
C16: Control group in day 16
C20: Control group in day 20
CAT: Catalase
MDA: Malonaldehyde
POD: Peroxidase
Pro: Proline
ROS: Reactive oxygen species
S16: Drought stressed plant in day 16
S20: Drought stressed plant in day 20
