# Supplementary material for: Physiological and proteomic analyses of the drought stress response in Amygdalus Mira (Koehne) Yü et Lu roots
Source: BMC Plant Biol. 2017 Feb 27;17:53. doi: 10.1186/s12870-017-1000-z (PMC5327565; doi:10.1186/s12870-017-1000-z)
Supplement: Additional file 1: Table S1. — Identification of drought-responsive proteins in Amygdalus mira (Koehne) Yü et Lu roots by mass spectrometry (MS) analysis. (DOC 2010 kb) [file 12870_2017_1000_MOESM1_ESM.doc]

**Table 2. Identification of drought-responsive proteins in *Amygdalus mira (Koehne) Yü et Lu* roots by mass spectrometry (MS) analysis**

| **Spot no.** | **Homologous protein** | **Species** | **Accession** | **Theor pI/Mr** | **Exper pI/Mr** | **score** | **C(%)** | **PN** | **Peptide sequence** | **Observed m/z** |
| --- | --- | --- | --- | --- | --- | --- | --- | --- | --- | --- |
| **Cytoskeleton dynamics** | | | | | | | | | | |
| 40 | profilin | *Malus domestica* | 4761584 | 5.41/14.06 | 6.1/16.2 | 56 | 69.23 | 5 | R.LGDYLIDQGL.- | 1106.5432 |
|  |  |  |  |  |  |  |  |  | K.FKPEEITAIMK.D | 1306.6764 |
|  |  |  |  |  |  |  |  |  | K.FKPEEITAIMK.D + Oxidation (M) | 1322.6418 |
|  |  |  |  |  |  |  |  |  | K.YMVIQGEGGAVIR.G | 1392.6990 |
|  |  |  |  |  |  |  |  |  | K.YMVIQGEGGAVIR.G + Oxidation (M) | 1408.6898 |
| 84 | actin 1 | *Physcomitrella patens* | 37038130 | 5.31/41.61 | 5.87/56 | 57 | 32.43 | 11 | K.AGFAGDDAPR.A | 976.4318 |
|  |  |  |  |  |  |  |  |  | R.GYSFTTTAER.E | 1132.5334 |
|  |  |  |  |  |  |  |  |  | R.HTGVMVGMGQK.D + 2 Oxidation (M) | 1176.5309 |
|  |  |  |  |  |  |  |  |  | K.DAYVGDEAQSK.R | 1182.5225 |
|  |  |  |  |  |  |  |  |  | R.AVFPSIVGRPR.H | 1198.5309 |
|  |  |  |  |  |  |  |  |  | K.SEYDESGPSIVHR.K | 1475.7069 |
|  |  |  |  |  |  |  |  |  | R.MSKEITALAPSSMK.I | 1493.6907 |
|  |  |  |  |  |  |  |  |  | R.LDLAGRDLTDALTK.I | 1501.6716 |
|  |  |  |  |  |  |  |  |  | K.IWHHTFYNELR.V | 1515.7040 |
|  |  |  |  |  |  |  |  |  | K.SYELPDGQVITIGAER.F | 1747.8440 |
|  |  |  |  |  |  |  |  |  | R.VAPEEHPVLLTEAPLNPK.A | 1954.0070 |
| 85 | ACT1 | *Actinidia deliciosa* | 149938964 | 5.31/41.66 | 5.2/48 | 549 | 67.57 | 19 | K.AGFAGDDAPR.A | 976.4113 |
|  |  |  |  |  |  |  |  |  | R.GYMFTTTAER.E | 1176.4913 |
|  |  |  |  |  |  |  |  |  | R.GYMFTTTAER.E + Oxidation (M) | 1192.4818 |
|  |  |  |  |  |  |  |  |  | R.AVFPSIVGRPR.H | 1198.6567 |
|  |  |  |  |  |  |  |  |  | K.DAYVGDEAQSKR.G | 1338.5747 |
|  |  |  |  |  |  |  |  |  | K.GEYDESGPSIVHR.K | 1445.6099 |
|  |  |  |  |  |  |  |  |  | K.IWHHTFYNELR.V | 1515.6913 |
|  |  |  |  |  |  |  |  |  | R.LDLAGRDLTDALMK.I | 1531.6813 |
|  |  |  |  |  |  |  |  |  | R.LDLAGRDLTDALMK.I + Oxidation (M) | 1547.6713 |
|  |  |  |  |  |  |  |  |  | K.NYELPDGQVITIGAER.F | 1774.8280 |
|  |  |  |  |  |  |  |  |  | K.LAYVALDYEQELETAK.S | 1855.8588 |
|  |  |  |  |  |  |  |  |  | K.YPIEHGIVSNWDDMEK.I | 1932.8165 |
|  |  |  |  |  |  |  |  |  | K.YPIEHGIVSNWDDMEK.I + Oxidation (M) | 1948.8108 |
|  |  |  |  |  |  |  |  |  | R.VAPEEHPVLLTEAPLNPK.A | 1953.9869 |
|  |  |  |  |  |  |  |  |  | K.DLYGNIVLSGGSTMFPGIADR.M | 2183.0168 |
|  |  |  |  |  |  |  |  |  | K.DLYGNIVLSGGSTMFPGIADR.M + Oxidation (M) | 2199.0149 |
|  |  |  |  |  |  |  |  |  | R.KDLYGNIVLSGGSTMFPGIADR.M | 2311.1145 |
|  |  |  |  |  |  |  |  |  | R.KDLYGNIVLSGGSTMFPGIADR.M + Oxidation (M) | 2327.1252 |
|  |  |  |  |  |  |  |  |  | R.TTGIVLDSGDGVSHTVPIYEGYALPHAILR.L | 3151.6316 |
| **Carbohydrate and nitrogen metabolism** | | | | | | | | | | |
| 20 | Succinyl-CoA ligase beta-chain family protein | *Populus trichocarpa* | 224065571 | 6.63/45.27 | 5.97/43.67 | 129 | 33.33 | 12 | R.NDSIEQVKK.L | 1060.5809 |
|  |  |  |  |  |  |  |  |  | K.LNFDDNAAFR.Q | 1182.6023 |
|  |  |  |  |  |  |  |  |  | R.LEGTNVDQGKR.I | 1216.6879 |
|  |  |  |  |  |  |  |  |  | K.GVAVASLHHVQK.A | 1245.6143 |
|  |  |  |  |  |  |  |  |  | K.AILVNIFGGIMK.C | 1275.7502 |
|  |  |  |  |  |  |  |  |  | K.FKNGFQGGVHIVK.T | 1430.8160 |
|  |  |  |  |  |  |  |  |  | R.LNIHEYQGAGLMGK.Y | 1530.8368 |
|  |  |  |  |  |  |  |  |  | K.FPDLIIKVPIDVFK.G | 1643.8564 |
|  |  |  |  |  |  |  |  |  | K.EIFALRDPTQEDPR.E | 1686.9047 |
|  |  |  |  |  |  |  |  |  | R.RLNIHEYQGAGLMGK.Y | 1686.9047 |
|  |  |  |  |  |  |  |  |  | K.MLGQILVTKQTGSQGK.I + Oxidation (M) | 1704.9711 |
|  |  |  |  |  |  |  |  |  | K.VEETAGKMLGQILVTK.Q | 1716.9825 |
| 26 | beta-hexosaminidase 2 | *Prunus persica* | 440355382 | 5.46/65.79 | 5.01/78 | 193 | 33.9 | 12 | K.RIVSSGYR.V | 937.5542 |
|  |  |  |  |  |  |  |  |  | R.LNEWRSR.I | 960.5820 |
|  |  |  |  |  |  |  |  |  | R.TIEAMSANK.L + Oxidation (M) | 980.5524 |
|  |  |  |  |  |  |  |  |  | K.NGGSWCGPFK.T | 1109.5298 |
|  |  |  |  |  |  |  |  |  | R.NSYGVRDMLR.T + Oxidation (M) | 1226.7000 |
|  |  |  |  |  |  |  |  |  | K.IVEFGLEHGVR.V | 1255.7249 |
|  |  |  |  |  |  |  |  |  | R.NPGMCNTFNPVA.- | 1321.6525 |
|  |  |  |  |  |  |  |  |  | R.ASAVAESLWSGNR.D | 1347.7164 |
|  |  |  |  |  |  |  |  |  | R.GVGAEPIQPLWCVR.N | 1581.8804 |
|  |  |  |  |  |  |  |  |  | R.LAAEPGTGHLNPLNPK.T | 1628.9331 |
|  |  |  |  |  |  |  |  |  | K.GSYGSNMHYSPADVTK.I | 1713.8229 |
|  |  |  |  |  |  |  |  |  | R.ASAVAESLWSGNRDEK.G | 1719.8940 |
|  |  |  |  |  |  |  |  |  | K.GSYGSNMHYSPADVTK.I + Oxidation (M) | 1729.8206 |
|  |  |  |  |  |  |  |  |  | R.TVVYWEDVLLDDNIK.V | 1822.0044 |
| 38 | alcohol dehydrogenase | *Prunus dulcis x Prunus persica* | 443410310 | 6.08/4.13 | 6.21/46.2 | 190 | 36.84 | 10 | K.GQHPLFPR.I | 951.5281 |
|  |  |  |  |  |  |  |  |  | R.IIGVDLNSNR.F | 1100.6149 |
|  |  |  |  |  |  |  |  |  | K.FGVNEFVNPK.D | 1150.6019 |
|  |  |  |  |  |  |  |  |  | K.GTFYGNYKPR.S | 1202.6124 |
|  |  |  |  |  |  |  |  |  | K.THPVNFLNER.T | 1226.6448 |
|  |  |  |  |  |  |  |  |  | K.KFGVNEFVNPK.D | 1278.6987 |
|  |  |  |  |  |  |  |  |  | K.AFDLMLSGQSIR.C | 1337.7097 |
|  |  |  |  |  |  |  |  |  | K.AFDLMLSGQSIR.C + Oxidation (M) | 1353.6539 |
|  |  |  |  |  |  |  |  |  | K.FITHSVPFAEINK.A | 1502.8132 |
|  |  |  |  |  |  |  |  |  | K.GSSVAIFGLGAVGLAAAEGAR.V | 1874.0354 |
| 59 | enolase | *Prunus armeniaca* | 63192024 | 5.13/15.86 | 5.45/34 | 143 | 78.57 | 7 | K.FRVPVEPY.- | 1006.5236 |
|  |  |  |  |  |  |  |  |  | K.AGWGVMASHR.S | 1071.5056 |
|  |  |  |  |  |  |  |  |  | K.AGWGVMASHR.S + Oxidation (M) | 1087.5028 |
|  |  |  |  |  |  |  |  |  | R.LAKYNQLLR.I | 1118.5474 |
|  |  |  |  |  |  |  |  |  | R.IEEELGAEAVYAGAK.F | 1549.7665 |
|  |  |  |  |  |  |  |  |  | K.VNQIGSVTESIEAVR.M | 1601.8369 |
|  |  |  |  |  |  |  |  |  | R.IEEELGAEAVYAGAKFR.V | 1852.9122 |
| 91 | Glyceraldehyde-3-phosphate dehydrogenase | *Medicago truncatula* | 355485087 | 8.93/41.68 | 6.46/58 | 60 | 23.08 | 6 | K.ASSHLKAGAK.K | 969.3962 |
|  |  |  |  |  |  |  |  |  | R.LQKNASYEDVK.A | 1294.4991 |
|  |  |  |  |  |  |  |  |  | R.VLDLVGHMALVGAQN.- + Oxidation (M) | 1552.7323 |
|  |  |  |  |  |  |  |  |  | K.LVSWYDNEWGYSNR.V | 1788.7303 |
|  |  |  |  |  |  |  |  |  | R.ATATEIPLPVQNSSSTGKTR.V | 2057.9922 |
|  |  |  |  |  |  |  |  |  | K.GILGYTDEDVVSNDFIGDSR.S | 2171.9282 |
| 92 | cytosolic aldolase | *Fragaria x ananassa* | 10645188 | 6.93/38.51 | 6.48/39.56 | 109 | 15.56 | 4 | K.LGEGAAESLHVK.D | 1210.6964 |
|  |  |  |  |  |  |  |  |  | K.ASPEVIAEYTVR.A | 1334.7443 |
|  |  |  |  |  |  |  |  |  | K.KASPEVIAEYTVR.A | 1462.8451 |
|  |  |  |  |  |  |  |  |  | K.IGPNEPSQLSINENANGLAR.Y | 2094.1431 |
| **Energy metabolism** | | | | | | | | | | |
| 1 | ATP synthase beta subunit | *Triticum aestivum* | 525291 | 5.56/59.2 | 5.4/56 | 490 | 50 | 17 | R.EGNDLYR.E | 866.4177 |
|  |  |  |  |  |  |  |  |  | K.IGLFGGAGVGK.T | 975.5768 |
|  |  |  |  |  |  |  |  |  | K.THDFLPIHR.E | 1135.6235 |
|  |  |  |  |  |  |  |  |  | K.VVDLLAPYQR.G | 1173.6833 |
|  |  |  |  |  |  |  |  |  | R.TIAMDGTEGLVR.G | 1262.6647 |
|  |  |  |  |  |  |  |  |  | R.TIAMDGTEGLVR.G + Oxidation (M) | 1278.6760 |
|  |  |  |  |  |  |  |  |  | K.AHGGFSVFAGVGER.T | 1390.7141 |
|  |  |  |  |  |  |  |  |  | R.VGLTGLTVAEHFR.D | 1399.7944 |
|  |  |  |  |  |  |  |  |  | K.VLNTGSPITVPVGR.A | 1409.8339 |
|  |  |  |  |  |  |  |  |  | K.TVLIMELINNVAK.A + Oxidation (M) | 1473.8813 |
|  |  |  |  |  |  |  |  |  | R.FTQANSEVSALLGR.I | 1492.7998 |
|  |  |  |  |  |  |  |  |  | R.LVLEVAQHLGENVVR.T | 1675.8734 |
|  |  |  |  |  |  |  |  |  | R.DAEGQDVLLFIDNIFR.F | 1864.9756 |
|  |  |  |  |  |  |  |  |  | R.EGNDLYREMIESGVIK.L + Oxidation (M) | 1869.0044 |
|  |  |  |  |  |  |  |  |  | R.QISELGIYPAVDPLDSTSR.M | 2061.0793 |
|  |  |  |  |  |  |  |  |  | R.EAPAFVEQATEQQILVTGIK.V | 2172.1897 |
|  |  |  |  |  |  |  |  |  | R.IPSAVGYQPTLATDLGGLQER.I | 2186.1814 |
| 13 | cytochrome P450 | *Atropa belladonna* | 58397706 | 8.93/9.0 | 5.6/21.78 | 13 | 25 | 1 | R.LHPPIPLLVRK.S | 1283.1101 |
| 46 | cytochrome c oxidase subunit | *Zea mays* | 195657267 | 4.35/18.3 | 4.57/35.7 | 114 | 62.5 | 5 | R.HCFTR.Y | 720.3069 |
|  |  |  |  |  |  |  |  |  | R.WNEQR.E | 732.3223 |
|  |  |  |  |  |  |  |  |  | R.YVEYHR.C | 866.3946 |
|  |  |  |  |  |  |  |  |  | R.FPTTNQTR.H | 964.4621 |
|  |  |  |  |  |  |  |  |  | K.IETAPADFR.F | 1019.4924 |
| 48 | PREDICTED: uncharacterized mitochondrial protein AtMg00820-like | *Glycine max* | 571551717 | 9.94/16.70 | 6.87/34 | 59 | 85.71 | 9 | K.AGVVNPR.L | 712.2539 |
|  |  |  |  |  |  |  |  |  | R.FDTSLFI.- | 842.4755 |
|  |  |  |  |  |  |  |  |  | K.TEHDALMK.N + Oxidation (M) | 960.5009 |
|  |  |  |  |  |  |  |  |  | K.ATLLQYNFR.S | 1125.5505 |
|  |  |  |  |  |  |  |  |  | R.SSRFDTSLFI.- | 1172.5684 |
|  |  |  |  |  |  |  |  |  | K.IALSNPTWFATMK.T | 1479.6859 |
|  |  |  |  |  |  |  |  |  | R.LHPTLLHAHFEPK.S | 1539.7231 |
|  |  |  |  |  |  |  |  |  | K.LAPSTAPIGCTWVFR.V | 1675.7572 |
|  |  |  |  |  |  |  |  |  | K.STKIALSNPTWFATMK.T + Oxidation (M) | 1811.8165 |
| 56 | ATP synthase beta subunit | *Asimina triloba* | 14717944 | 8.24/4.10 | 5.89/66.09 | 58 | 34.21 | 12 | K.FSIFETGIK.V | 1041.5331 |
|  |  |  |  |  |  |  |  |  | K.VVDLLAPYR.R | 1045.5687 |
|  |  |  |  |  |  |  |  |  | R.SAPAFTQLDTK.F | 1178.6250 |
|  |  |  |  |  |  |  |  |  | K.VVDLLAPYRR.G | 1201.5422 |
|  |  |  |  |  |  |  |  |  | K.MPYIYNALVVK.G | 1310.6410 |
|  |  |  |  |  |  |  |  |  | K.AHGGVSVFGAVGER.T | 1342.7040 |
|  |  |  |  |  |  |  |  |  | K.TVLIMELINNIAK.A + Oxidation (M) | 1487.7015 |
|  |  |  |  |  |  |  |  |  | R.VGLTALTMAEYFR.D + Oxidation (M) | 1487.7015 |
|  |  |  |  |  |  |  |  |  | R.VRAVAMSATDGLMR.G + Oxidation (M) | 1493.7152 |
|  |  |  |  |  |  |  |  |  | R.GKMPYIYNALVVK.G | 1495.7217 |
|  |  |  |  |  |  |  |  |  | K.MPYIYNALVVKGR.D | 1523.7587 |
|  |  |  |  |  |  |  |  |  | K.VALVYGQMNEPPGAR.M + Oxidation (M) | 1617.7865 |
| 47 | Biotin carboxylase | *Medicago truncatula* | 355524630 | 7.94/59.03 | 6.56/48.02 | 65 | 27.78 | 12 | K.LLQQAK.S | 700.3158 |
|  |  |  |  |  |  |  |  |  | K.MVLVNK.V | 703.3257 |
|  |  |  |  |  |  |  |  |  | R.HCGALR.A | 713.3224 |
|  |  |  |  |  |  |  |  |  | K.YIQNPR.H | 790.4031 |
|  |  |  |  |  |  |  |  |  | K.LIVWAPTR.E | 955.5477 |
|  |  |  |  |  |  |  |  |  | K.YGNVVHFGER.D | 1177.5487 |
|  |  |  |  |  |  |  |  |  | R.HIEFQVLADK.Y | 1199.5952 |
|  |  |  |  |  |  |  |  |  | R.ITAYLPSGGPFVR.M | 1377.7195 |
|  |  |  |  |  |  |  |  |  | R.GSFYFMEMNTR.I + 2 Oxidation (M) | 1414.5848 |
|  |  |  |  |  |  |  |  |  | K.LLEEAPSPALTPELR.K | 1635.8552 |
|  |  |  |  |  |  |  |  |  | K.SEAAAAFGNDGVYLEK.Y | 1641.7412 |
|  |  |  |  |  |  |  |  |  | R.DHGVNFIGPNPDSIRVMGDK.A + Oxidation (M) | 2184.0525 |
| 22 | Cobalt import ATP-binding protein cbiO, putative | *Ricinus communis* | 223525527 | 6.80/36.05 | 4.56/32.78 | 52 | 34.38 | 11 | M.AVVECK.Q | 705.3961 |
|  |  |  |  |  |  |  |  |  | K.QMDGSR.V + Oxidation (M) | 709.3517 |
|  |  |  |  |  |  |  |  |  | R.VTGDPAR.V | 715.4456 |
|  |  |  |  |  |  |  |  |  | K.ECEER.G | 722.3466 |
|  |  |  |  |  |  |  |  |  | K.VSDGQRR.R | 817.4300 |
|  |  |  |  |  |  |  |  |  | R.VTGDPARVAVR.A | 1140.5826 |
|  |  |  |  |  |  |  |  |  | R.VAVRALNNGWAAGR.L | 1454.7733 |
|  |  |  |  |  |  |  |  |  | M.AVVECKQATIETK.G | 1476.7759 |
|  |  |  |  |  |  |  |  |  | R.DELIKVLDVDLSWR.M | 1700.8386 |
|  |  |  |  |  |  |  |  |  | R.EVAFAGFEVPIQMDVSAEK.M + Oxidation (M) | 2083.0322 |
|  |  |  |  |  |  |  |  |  | R.REVAFAGFEVPIQMDVSAEK.M + Oxidation (M) | 2239.1609 |
| **Transcription and translation** | | | | | | | | | | |
| 3 | ORF; able to induce HR-like lesions | *Nicotiana tabacum* | 1762941 | 5.02/8.19 | 5.34/54.67 | 25 | 28.57 | 2 | -.MEEKIQR.M | 933.2300 |
|  |  |  |  |  |  |  |  |  | K.IQRMEEK.S | 933.2300 |
| 8 | WRKY transcription factor 19 | *Syagrus vermicularis* | 262088225 | 8.2/1.18 | 6.78/26.54 | 15 | 100 | 1 | R.NYYRCSSE.- | 1078.142 |
| 9 | WRKY transcription factor 19 | *Syagrus vermicularis* | 262088225 | 8.2/1.18 | 4.78/27.35 | 17 | 100 | 1 | R.NYYRCSSE.- | 1078.1283 |
| 19 | dead box ATP-dependent RNA helicase, putative | *Ricinus communis* | 223528461 | 9.84/88.27 | 5.2/58.54 | 53 | 24.05 | 15 | R.ALILSPTR.D | 870.5536 |
|  |  |  |  |  |  |  |  |  | K.ASQMSHMKSK.G | 1134.6798 |
|  |  |  |  |  |  |  |  |  | R.SEIKSLEQVR.K | 1188.6415 |
|  |  |  |  |  |  |  |  |  | K.TKPLPAKESIR.R | 1239.6508 |
|  |  |  |  |  |  |  |  |  | K.AEPLVSSKAELK.H | 1271.6608 |
|  |  |  |  |  |  |  |  |  | R.DLALQTLKFTK.E | 1277.7274 |
|  |  |  |  |  |  |  |  |  | K.KQNFMPNANVR.S | 1318.6759 |
|  |  |  |  |  |  |  |  |  | R.LDVDTKISPDLK.T | 1343.7302 |
|  |  |  |  |  |  |  |  |  | R.VKDLPHEGIHPIFK.N | 1629.8950 |
|  |  |  |  |  |  |  |  |  | K.TMPIILSGSDVVAMAR.T | 1660.9471 |
|  |  |  |  |  |  |  |  |  | R.LMHHLSEVDDMSLR.T | 1682.9103 |
|  |  |  |  |  |  |  |  |  | K.QHVSQGGARALILSPTR.D | 1790.9507 |
|  |  |  |  |  |  |  |  |  | R.KTMPIILSGSDVVAMAR.T + 2 Oxidation (M) | 1820.9462 |
|  |  |  |  |  |  |  |  |  | R.VMMKINEAVANGETIYGR.F + Oxidation (M) | 2012.0247 |
|  |  |  |  |  |  |  |  |  | K.TMPIILSGSDVVAMARTGSGK.T | 2090.9846 |
| 28 | PREDICTED: ethylene-responsive transcription factor 1A-like | *Glycine max* | 356563364 | 5.6/27.25 | 5.2/31.56 | 60 | 40 | 9 | K.FAAEIR.D | 706.4007 |
|  |  |  |  |  |  |  |  |  | R.AAYRMR.G + Oxidation (M) | 783.3718 |
|  |  |  |  |  |  |  |  |  | R.SSSPESMAAAAPKR.K + Oxidation (M) | 1405.7083 |
|  |  |  |  |  |  |  |  |  | R.SDSYESDLALLDSIR.R | 1683.8640 |
|  |  |  |  |  |  |  |  |  | R.SDSYESDLALLDSIRR.H | 1839.9321 |
|  |  |  |  |  |  |  |  |  | R.ALLNFPLRVNSGEPDPVR.V | 1994.0125 |
|  |  |  |  |  |  |  |  |  | R.HLLGESELIFGAPNFGSGR.S | 2001.1775 |
|  |  |  |  |  |  |  |  |  | -.MYGRSDSYESDLALLDSIR.R + Oxidation (M) | 2207.1812 |
|  |  |  |  |  |  |  |  |  | K.VMVVGTVQEQVGSQVVECTR.G + Oxidation (M) | 2221.1594 |
| 29 | histone-like protein | *Fritillaria liliacea* | 15281602 | 10.87/17.75 | 6.28/21.67 | 59 | 64.71 | 11 | K.TAGRPAK.A | 700.2979 |
|  |  |  |  |  |  |  |  |  | K.AAKTSVK.A | 704.3342 |
|  |  |  |  |  |  |  |  |  | K.TVALKGK.T | 716.3369 |
|  |  |  |  |  |  |  |  |  | K.APAVNMK.S + Oxidation (M) | 746.3916 |
|  |  |  |  |  |  |  |  |  | K.AAPVALKK.A | 797.3569 |
|  |  |  |  |  |  |  |  |  | K.SHLPANFK.K | 913.4074 |
|  |  |  |  |  |  |  |  |  | K.TAGRPAKAAK.T | 970.4628 |
|  |  |  |  |  |  |  |  |  | K.LLLVQLRK.L | 982.4418 |
|  |  |  |  |  |  |  |  |  | K.SAAVKPKAPAVNMK.S + Oxidation (M) | 1427.7526 |
|  |  |  |  |  |  |  |  |  | -.EMISEAIASLKER.T | 1476.7416 |
|  |  |  |  |  |  |  |  |  | K.NSYKISAKPTPAAKPK.S | 1700.8090 |
| 49 | retrotransposon protein, putative, Ty3-gypsy subclass | *Oryza sativa Japonica Group* | 31430228 | 8.54/180.36 | 4.60/16.56 | 57 | 17.83 | 25 | R.LVAGFAK.E | 705.3674 |
|  |  |  |  |  |  |  |  |  | -.MQDAAR.E + Oxidation (M) | 707.3295 |
|  |  |  |  |  |  |  |  |  | K.KDGSMR.M + Oxidation (M) | 709.3254 |
|  |  |  |  |  |  |  |  |  | K.SYADTR.R | 712.3052 |
|  |  |  |  |  |  |  |  |  | K.TPQTNR.T | 716.3281 |
|  |  |  |  |  |  |  |  |  | K.NEFRR.L | 721.3419 |
|  |  |  |  |  |  |  |  |  | K.LYAKFSK.C | 856.4944 |
|  |  |  |  |  |  |  |  |  | R.KSTRPPGK.K | 870.5110 |
|  |  |  |  |  |  |  |  |  | R.VCAMDFSK.D + Oxidation (M) | 973.4976 |
|  |  |  |  |  |  |  |  |  | K.QIRELQEK.G | 1043.5983 |
|  |  |  |  |  |  |  |  |  | R.GGSSFGEFMR.T | 1074.5424 |
|  |  |  |  |  |  |  |  |  | R.GGSSFGEFMR.T + Oxidation (M) | 1090.4948 |
|  |  |  |  |  |  |  |  |  | R.EDLPGMPPDR.D | 1126.5049 |
|  |  |  |  |  |  |  |  |  | R.IVCLHGVPKR.I | 1178.5593 |
|  |  |  |  |  |  |  |  |  | R.TNQVLEDMLR.V + Oxidation (M) | 1234.6324 |
|  |  |  |  |  |  |  |  |  | K.VEAVTEWKAPK.F | 1257.5015 |
|  |  |  |  |  |  |  |  |  | K.MSPNEALFGRR.C | 1277.6652 |
|  |  |  |  |  |  |  |  |  | -.MQDAAREAFLR.L | 1307.6202 |
|  |  |  |  |  |  |  |  |  | R.TPLMWTETGER.A | 1320.5458 |
|  |  |  |  |  |  |  |  |  | R.GPMIGQDHESFR.S | 1373.6123 |
|  |  |  |  |  |  |  |  |  | K.DGSMRMCVDYR.S + Oxidation (M) | 1405.7499 |
|  |  |  |  |  |  |  |  |  | R.YAIGDLANEEEKIDK.F | 1707.7069 |
|  |  |  |  |  |  |  |  |  | K.EKLTSSPILVLPDIWK.D | 1838.8560 |
|  |  |  |  |  |  |  |  |  | K.AEHQRPAGLLQPLRIPEWK.W | 2239.0288 |
|  |  |  |  |  |  |  |  |  | R.HKGATSSGWKPPIVATNRPAAPR.N | 2399.1606 |
| 54 | ribosomal protein S18 | *Floydiella terrestris* | 304322971 | 11.52/26.49 | 5.5/48.39 | 31 | 17.39 | 3 | K.KESILPEVPK.K | 1139.6926 |
|  |  |  |  |  |  |  |  |  | K.ESILPEVPKK.A | 1139.6926 |
|  |  |  |  |  |  |  |  |  | -.MNQTQRPKK.E + Oxidation (M) | 1146.5826 |
| 57 | Mitochondrial HSO70 2 isoform 2 | *Theobroma cacao* | 508706435 | 5.71/74.42 | 5.2/67.45 | 179 | 74.07 | 17 | K.HLNITLTR.S | 967.5616 |
|  |  |  |  |  |  |  |  |  | K.VIENSEGAR.T | 974.4880 |
|  |  |  |  |  |  |  |  |  | K.EMGMVPYK.I + 2 Oxidation (M) | 986.4669 |
|  |  |  |  |  |  |  |  |  | K.SLNEYRDK.I | 1024.5116 |
|  |  |  |  |  |  |  |  |  | K.GELLVGTPAKR.Q | 1140.6686 |
|  |  |  |  |  |  |  |  |  | R.TTPSVVAFNQK.G | 1191.6281 |
|  |  |  |  |  |  |  |  |  | K.FESLVNHLIER.T | 1356.7125 |
|  |  |  |  |  |  |  |  |  | R.KAMEGENVDEIK.A | 1362.6694 |
|  |  |  |  |  |  |  |  |  | K.DVDEVLLVGGMTR.V | 1403.7112 |
|  |  |  |  |  |  |  |  |  | K.DVDEVLLVGGMTR.V + Oxidation (M) | 1419.7112 |
|  |  |  |  |  |  |  |  |  | R.QAVTNPTNTVFGTK.R | 1477.7607 |
|  |  |  |  |  |  |  |  |  | K.AVITVPAYFNDAQR.Q | 1564.7970 |
|  |  |  |  |  |  |  |  |  | R.SKFESLVNHLIER.T | 1571.8398 |
|  |  |  |  |  |  |  |  |  | K.SQVFSTAADNQTQVGIK.V | 1793.8993 |
|  |  |  |  |  |  |  |  |  | R.IINEPTAAALSYGMNNK.E | 1806.9012 |
|  |  |  |  |  |  |  |  |  | K.GVNPDEAVAMGAAIQGGILR.G | 1938.9906 |
|  |  |  |  |  |  |  |  |  | K.GVNPDEAVAMGAAIQGGILR.G + Oxidation (M) | 1954.9969 |
| 58 | PREDICTED: ribonuclease R-like | *Cucumis sativus* | 449474891 | 5.88/60.67 | 6.15/110.5 | 56 | 16.98 | 5 | K.HIEELHNLYK.T | 1295.6565 |
|  |  |  |  |  |  |  |  |  | K.GVIASVTGFGFFVR.L | 1456.8589 |
|  |  |  |  |  |  |  |  |  | K.LIEECMILANISAAR.F + Oxidation (M) | 1719.8345 |
|  |  |  |  |  |  |  |  |  | R.VDLRDLPLVTIDGEDAR.D | 1896.9323 |
|  |  |  |  |  |  |  |  |  | R.FDQVGQRLIGESGGQTYR.L | 2010.9896 |
| 61 | Elongation factor Tu family protein | *Populus trichocarpa* | 566159427 | 7.15/49.03 | 6.45/44 | 59 | 22.73 | 9 | R.TADITGK.V | 705.3489 |
|  |  |  |  |  |  |  |  |  | R.NPNSKR.L | 715.2889 |
|  |  |  |  |  |  |  |  |  | -.MASLALR.N | 761.3182 |
|  |  |  |  |  |  |  |  |  | R.TVGAGVVSK.V | 817.3399 |
|  |  |  |  |  |  |  |  |  | K.EHILLAR.Q | 851.4575 |
|  |  |  |  |  |  |  |  |  | K.VELPENVK.M | 927.4042 |
|  |  |  |  |  |  |  |  |  | K.AIAFDEIDK.A | 1021.4573 |
|  |  |  |  |  |  |  |  |  | K.FPGDEIPIVR.G | 1142.5540 |
|  |  |  |  |  |  |  |  |  | K.LMDAVDEYIPDPVR.Q + Oxidation (M) | 1648.6963 |
| 64 | putative XH domain family protein | *Zea mays* | 413947404 | 5.09/78.76 | 6.69/29.6 | 53 | 30.43 | 17 | K.EDVLAR.M | 702.3180 |
|  |  |  |  |  |  |  |  |  | R.IDLESK.K | 704.2961 |
|  |  |  |  |  |  |  |  |  | R.FEREK.K | 708.3054 |
|  |  |  |  |  |  |  |  |  | K.TLDNEK.Q | 719.3086 |
|  |  |  |  |  |  |  |  |  | K.EFEMR.D + Oxidation (M) | 727.3011 |
|  |  |  |  |  |  |  |  |  | R.ADADVLK.L | 731.3237 |
|  |  |  |  |  |  |  |  |  | R.TAIGVKR.M | 744.3080 |
|  |  |  |  |  |  |  |  |  | K.KEFEMR.D | 839.3263 |
|  |  |  |  |  |  |  |  |  | R.LNGTLQVMK.H | 1003.3783 |
|  |  |  |  |  |  |  |  |  | K.ISTENANDR.K | 1019.3678 |
|  |  |  |  |  |  |  |  |  | K.YGKDEYEIK.A | 1144.4774 |
|  |  |  |  |  |  |  |  |  | K.ANVDTAGSSSVAAK.V | 1277.6074 |
|  |  |  |  |  |  |  |  |  | K.ERESNDELQEAR.K | 1475.6306 |
|  |  |  |  |  |  |  |  |  | K.AKANVDTAGSSSVAAK.V | 1476.6261 |
|  |  |  |  |  |  |  |  |  | K.LHEAYNEEMRNLHR.R | 1811.7367 |
|  |  |  |  |  |  |  |  |  | K.RMGELDEKPFQNACK.T + Oxidation (M) | 1838.7776 |
|  |  |  |  |  |  |  |  |  | K.AKYDNSELELASIEQQR.A | 1993.8143 |
| 66 | signal recognition particle 54 kDa subunit | *Arabidopsis thaliana* | 515681 | 9.02/54.71 | 5.7/27.8 | 57 | 36.73 | 15 | K.GGGALSAVAATK.S | 1002.4901 |
|  |  |  |  |  |  |  |  |  | R.NMNAQHMSK.V | 1060.5063 |
|  |  |  |  |  |  |  |  |  | K.IAVEGVDTFK.K | 1078.5680 |
|  |  |  |  |  |  |  |  |  | -.MVLAELGGGITR.A | 1216.6606 |
|  |  |  |  |  |  |  |  |  | K.QEASLFEEMR.Q | 1239.6758 |
|  |  |  |  |  |  |  |  |  | R.EVMEMLEEYK.R | 1300.7522 |
|  |  |  |  |  |  |  |  |  | K.ENCDLIIVDTSGR.H | 1491.7480 |
|  |  |  |  |  |  |  |  |  | R.QVREVMEMLEEYK.R | 1683.8096 |
|  |  |  |  |  |  |  |  |  | R.QVREVMEMLEEYK.R + 2 Oxidation (M) | 1715.8250 |
|  |  |  |  |  |  |  |  |  | K.QMGSGKDMMGMFGGGDK.- + 2 Oxidation (M) | 1765.8477 |
|  |  |  |  |  |  |  |  |  | K.EVFSMLPGISAEMMPK.G + Oxidation (M) | 1782.8654 |
|  |  |  |  |  |  |  |  |  | K.LQEVVPKDQQPELLEK.L | 1892.9629 |
|  |  |  |  |  |  |  |  |  | R.IMYDQFQNILNMGPLK.E | 1924.9277 |
|  |  |  |  |  |  |  |  |  | K.QIGGMGGLQSLMKQMGSGK.D + 3 Oxidation (M) | 1955.9056 |
|  |  |  |  |  |  |  |  |  | R.NMNAQHMSKVLPPQMLK.Q | 1967.0219 |
| 69 | Transcription-repair-coupling factor | *Triticum urartu* | 474420269 | 9.05/78.07 | 5.56/30.68 | 57 | 26.09 | 15 | K.RPRNLSK.L | 870.5177 |
|  |  |  |  |  |  |  |  |  | R.LICGDVGFGK.T | 1065.4965 |
|  |  |  |  |  |  |  |  |  | K.GMAARSSLGHR.V + Oxidation (M) | 1158.5465 |
|  |  |  |  |  |  |  |  |  | K.RGGQVFYVVPR.I | 1277.6771 |
|  |  |  |  |  |  |  |  |  | R.DASLMSTPPPER.V | 1300.5781 |
|  |  |  |  |  |  |  |  |  | R.EGFTYLFYTDK.S | 1383.6541 |
|  |  |  |  |  |  |  |  |  | K.ASIDVLTLSATPIPR.T | 1553.7577 |
|  |  |  |  |  |  |  |  |  | R.SGREGFTYLFYTDK.S | 1683.7979 |
|  |  |  |  |  |  |  |  |  | R.DASTEWLGGRQCAEK.H | 1707.7987 |
|  |  |  |  |  |  |  |  |  | R.KQGYPGEWATPALAPR.D | 1741.8284 |
|  |  |  |  |  |  |  |  |  | K.NIQLAMEKFSCGEIK.I + Oxidation (M) | 1783.8535 |
|  |  |  |  |  |  |  |  |  | R.TIVHDLFRPYCSRK.I | 1791.7112 |
|  |  |  |  |  |  |  |  |  | K.MVVNLMELYLQRMR.Q | 1795.8616 |
|  |  |  |  |  |  |  |  |  | R.ETPMDRLICGDVGFGK.T + Oxidation (M) | 1810.8390 |
|  |  |  |  |  |  |  |  |  | K.DYRPIGLTHLFAKLVVK.I | 1969.8903 |
| 23 | HAT family dimerization domain containing protein | *Medicago truncatula* | 355489993 | 8.48/96.14 | 4.58/29.69 | 46 | 16.87 | 8 | K.SLDDVDIIR.T | 1045.6050 |
|  |  |  |  |  |  |  |  |  | R.LTCSLDCTR.Y | 1125.6101 |
|  |  |  |  |  |  |  |  |  | R.TITNLHHYR.A | 1154.6479 |
|  |  |  |  |  |  |  |  |  | R.GHDEKTTSLNK.G | 1229.6486 |
|  |  |  |  |  |  |  |  |  | K.GPMQPNLSSFPR.T + Oxidation (M) | 1346.6890 |
|  |  |  |  |  |  |  |  |  | R.EQLDTYVLQMK.R | 1367.7747 |
|  |  |  |  |  |  |  |  |  | K.MESFKFAFILK.L + Oxidation (M) | 1376.8553 |
|  |  |  |  |  |  |  |  |  | R.AYILKGPMQPNLSSFPR.T | 1918.9712 |
| 76 | ER-binding protein | *Malus pumila* | 57639078 | 5.14/73.57 | 4.82/70.53 | 373 | 34.84 | 18 | R.IMEYFIK.L | 943.4797 |
|  |  |  |  |  |  |  |  |  | R.IMEYFIK.L + Oxidation (M) | 959.4776 |
|  |  |  |  |  |  |  |  |  | K.NQAAVNPER.T | 998.4822 |
|  |  |  |  |  |  |  |  |  | R.ALSSQHQVR.V | 1025.5281 |
|  |  |  |  |  |  |  |  |  | K.DAGVIAGLNVAR.I | 1155.6224 |
|  |  |  |  |  |  |  |  |  | K.DYFDGKEPNK.G | 1212.5349 |
|  |  |  |  |  |  |  |  |  | K.MKETAEAFLGK.K | 1224.5887 |
|  |  |  |  |  |  |  |  |  | R.FEELNNDLFR.K | 1296.5846 |
|  |  |  |  |  |  |  |  |  | R.NSLETYVYNMK.N | 1361.6154 |
|  |  |  |  |  |  |  |  |  | R.QIDEIVLVGGSTR.I | 1386.7208 |
|  |  |  |  |  |  |  |  |  | R.ARFEELNNDLFR.K | 1523.7184 |
|  |  |  |  |  |  |  |  |  | K.NGHVEIIANDQGNR.I | 1536.7123 |
|  |  |  |  |  |  |  |  |  | K.VFSPEEVSAMILTK.M + Oxidation (M) | 1566.6936 |
|  |  |  |  |  |  |  |  |  | K.DAVVTVPAYFNDAQR.Q | 1665.7772 |
|  |  |  |  |  |  |  |  |  | R.IINEPTAAAIAYGLDKK.G | 1787.9434 |
|  |  |  |  |  |  |  |  |  | K.IKDAVVTVPAYFNDAQR.Q | 1906.9525 |
|  |  |  |  |  |  |  |  |  | R.VEIESLFDGVDFSEPLTR.A | 2052.9585 |
|  |  |  |  |  |  |  |  |  | K.SQVFTTYQDQQTTVSIQVFEGER.S | 2691.2429 |
| 79 | RNA-binding KH domain-containing protein | *Theobroma cacao* | 508719137 | 4.88/53.64 | 5.20/69.7 | 128 | 20.93 | 6 | K.ILDGPPGTTER.A | 1155.5691 |
|  |  |  |  |  |  |  |  |  | R.ASGATVTIQETR.G | 1233.6119 |
|  |  |  |  |  |  |  |  |  | K.WPGWPGESVFR.M | 1317.6006 |
|  |  |  |  |  |  |  |  |  | R.RASGATVTIQETR.G | 1389.7046 |
|  |  |  |  |  |  |  |  |  | K.KWPGWPGESVFR.M | 1445.6934 |
|  |  |  |  |  |  |  |  |  | R.IVDGLDGDSSHAPTAVGTK.V | 1839.8317 |
| 89 | NAC transcription factor 1 | *Salvia miltiorrhiza* | 532021153 | 4.39/21.66 | 4.7/30.68 | 227 | 52.63 | 5 | K.LGMKPIPGVSR.V | 1154.7104 |
|  |  |  |  |  |  |  |  |  | K.LGMKPIPGVSR.V + Oxidation (M) | 1170.7053 |
|  |  |  |  |  |  |  |  |  | K.DIELVMTQAGVSR.A | 1418.7798 |
|  |  |  |  |  |  |  |  |  | K.NILFVISKPDVFK.S | 1519.9415 |
|  |  |  |  |  |  |  |  |  | K.IEDLSSQLQTQAAEQFK.A | 1936.0448 |
| **Transport** | | | | | | | | | | |
| 4 | clathrin assembly protein | *Zea mays* | 195656105 | 10.67/35.57 | 5.3/40.45 | 50 | 29.41 | 10 | R.TILPIR.A | 712.3264 |
|  |  |  |  |  |  |  |  |  | R.LADEDR.V | 718.3707 |
|  |  |  |  |  |  |  |  |  | K.CLVALR.V | 731.3668 |
|  |  |  |  |  |  |  |  |  | -.MGGLKLR.R + Oxidation (M) | 790.3851 |
|  |  |  |  |  |  |  |  |  | R.AEDVVLR.R | 801.4127 |
|  |  |  |  |  |  |  |  |  | R.DAAGSASAR.T | 805.4042 |
|  |  |  |  |  |  |  |  |  | K.CLVALRVLLAR.G | 1283.7056 |
|  |  |  |  |  |  |  |  |  | R.GIAARVQEMGER.L + Oxidation (M) | 1332.7257 |
|  |  |  |  |  |  |  |  |  | R.GAFILRDQLLAALAR.H | 1627.8578 |
|  |  |  |  |  |  |  |  |  | R.QAPPPPPSGPQPNALIWEAVR.L | 2222.1311 |
| 5 | UDP-L-arabinose mutase-like protein | *Selaginella moellendorffii* | 300170356 | 6.26/4.39 | 5.94/38.43 | 147 | 38.46 | 12 | R.GYPFSLR.E | 839.4547 |
|  |  |  |  |  |  |  |  |  | K.ASNPFVNLK.K | 989.5495 |
|  |  |  |  |  |  |  |  |  | K.DINALEQHIK.N | 1180.6489 |
|  |  |  |  |  |  |  |  |  | K.VASPPPPPPAVVK.S | 1255.6257 |
|  |  |  |  |  |  |  |  |  | K.DELDIVIPTIR.N | 1283.7407 |
|  |  |  |  |  |  |  |  |  | K.VPEGFDYELYNR.N | 1501.7236 |
|  |  |  |  |  |  |  |  |  | K.YIFTIDDDCFVAK.D | 1606.7737 |
|  |  |  |  |  |  |  |  |  | R.ELIGPAMYFGLMGDGQPIGR.Y | 2122.0774 |
|  |  |  |  |  |  |  |  |  | R.ELIGPAMYFGLMGDGQPIGR.Y + Oxidation (M) | 2138.0784 |
|  |  |  |  |  |  |  |  |  | R.ELIGPAMYFGLMGDGQPIGR.Y + 2 Oxidation (M) | 2154.0676 |
|  |  |  |  |  |  |  |  |  | K.LADAMVTWTESWEELTSNK.I | 2211.1343 |
|  |  |  |  |  |  |  |  |  | K.GIFWQEEIIPFFQSAVLSK.E | 2239.1631 |
| 17 | DYNAMIN-LIKE protein 5 | *Populus trichocarpa* | 224111434 | 8.08/68.74 | 5.79/58.90 | 52 | 13.11 | 4 | R.KSIALTEELK.R | 1131.6821 |
|  |  |  |  |  |  |  |  |  | K.HLETVIRQR.I | 1151.5912 |
|  |  |  |  |  |  |  |  |  | R.RLIDGSISYFK.G | 1298.7375 |
|  |  |  |  |  |  |  |  |  | R.RIGSNVSAYIGMVCDTLR.N + Oxidation (M) | 2028.0311 |
| 83 | alpha-tubulin 4 | *Gossypium hirsutum* | 37529490 | 4.93/49.57 | 5.31/48.34 | 310 | 60 | 21 | K.EDAANNFAR.G | 1007.5004 |
|  |  |  |  |  |  |  |  |  | K.FDLMYTKR.A + Oxidation (M) | 1089.6565 |
|  |  |  |  |  |  |  |  |  | K.EIVDLCLDR.I | 1132.6208 |
|  |  |  |  |  |  |  |  |  | K.YMACCLMYR.G | 1267.5679 |
|  |  |  |  |  |  |  |  |  | K.YMACCLMYR.G + Oxidation (M) | 1283.5651 |
|  |  |  |  |  |  |  |  |  | K.YMACCLMYR.G + 2 Oxidation (M) | 1299.5664 |
|  |  |  |  |  |  |  |  |  | R.QLFHPEQLISGK.E | 1396.8209 |
|  |  |  |  |  |  |  |  |  | R.LVSQVISSLTASLR.F | 1473.9296 |
|  |  |  |  |  |  |  |  |  | R.TIQFVDWCPTGFK.C | 1598.8463 |
|  |  |  |  |  |  |  |  |  | R.SLDIERPTYTNLNR.L | 1691.9614 |
|  |  |  |  |  |  |  |  |  | R.AVFVDLEPTVIDEVR.T | 1701.9904 |
|  |  |  |  |  |  |  |  |  | R.IHFMLSSYAPVISAEK.A | 1793.0184 |
|  |  |  |  |  |  |  |  |  | R.IHFMLSSYAPVISAEK.A + Oxidation (M) | 1809.0197 |
|  |  |  |  |  |  |  |  |  | R.AVCMISNSTSVAEVFSR.I | 1857.9806 |
|  |  |  |  |  |  |  |  |  | R.AVCMISNSTSVAEVFSR.I + Oxidation (M) | 1873.9922 |
|  |  |  |  |  |  |  |  |  | K.CGINYQPPTVVPGGDLAK.V | 1886.0415 |
|  |  |  |  |  |  |  |  |  | K.TVGGGDDAFNTFFSETGAGK.H | 1977.9902 |
|  |  |  |  |  |  |  |  |  | R.AFVHWYVGEGMEEGEFSEAR.E | 2330.1497 |
|  |  |  |  |  |  |  |  |  | R.AFVHWYVGEGMEEGEFSEAR.E + Oxidation (M) | 2346.1699 |
|  |  |  |  |  |  |  |  |  | R.QLFHPEQLISGKEDAANNFAR.G | 2385.3369 |
|  |  |  |  |  |  |  |  |  | R.FDGALNVDVTEFQTNLVPYPR.I | 2395.3252 |
| **Inducer** | | | | | | | | | | |
| 10 | induced stolon tip protein PJ-1 | *Capsicum annuum* | 40287510 | 5.36/2.81 | 4.8/26.56 | 16 | 100 | 1 | -.MECCGPYR.G | 1072.1094 |
| 35 | induced stolon tip protein PJ-1 | *Capsicum annuum* | 40287510 | 5.36/2.81 | 4.8/82.67 | 15 | 100 | 1 | -.MECCGPYR.G | 1072.1129 |
| 37 | induced stolon tip protein PJ-1 | *Capsicum annuum* | 40287510 | 5.36/2.81 | 6.8/49.04 | 15 | 100 | 1 | M.VLGFTILFVKR.R | 1293.1005 |
| **Stress and defense** | | | | | | | | | | |
| 2 | superoxide dismutase [Cu-Zn] 1 | *Solanum lycopersicum* | 350537277 | 5.83/15.30 | 5.35/50.3 | 147 | 26.67 | 2 | R.AVVVHADPDDLGK.G | 1335.6287 |
|  |  |  |  |  |  |  |  |  | K.QIPLTGPQSIIGR.A | 1379.7338 |
| 6 | PREDICTED: arginase 1, mitochondrial-like isoform X3 | *Glycine max* | 571435280 | 6.46/30.36 | 5.52/35.45 | 62 | 25 | 4 | R.GICYMPR.L + Oxidation (M) | 912.4586 |
|  |  |  |  |  |  |  |  |  | R.DRHFLENLK.L | 1171.6615 |
|  |  |  |  |  |  |  |  |  | K.FGVEQYEMR.T + Oxidation (M) | 1174.5532 |
|  |  |  |  |  |  |  |  |  | R.VLTDVGDVPIQEIR.D | 1553.8868 |
| 11 | PREDICTED: 1,2-dihydroxy-3-keto-5-methylthiopentene dioxygenase 3-like isoform X1 | *Cicer arietinum* | 502119596 | 5.86/28.48 | 5.34/68 | 56 | 29.17 | 5 | K.IREER.G | 702.3649 |
|  |  |  |  |  |  |  |  |  | R.LLWRR.M | 743.3703 |
|  |  |  |  |  |  |  |  |  | R.FQCAER.N | 810.3933 |
|  |  |  |  |  |  |  |  |  | R.YAVAGSGYFDVR.D | 1304.6591 |
|  |  |  |  |  |  |  |  |  | K.SFFEEHLHTDEEIR.Y | 1788.8607 |
| 12 | NBS-LRR-like protein, partial | *Panicum virgatum* | 390429696 | 9.26/36.79 | 5.45/25 | 57 | 40.63 | 13 | K.MLTGLR.K + Oxidation (M) | 706.4164 |
|  |  |  |  |  |  |  |  |  | K.SIDSCK.V | 709.3755 |
|  |  |  |  |  |  |  |  |  | K.MMFLR.V + Oxidation (M) | 713.3481 |
|  |  |  |  |  |  |  |  |  | K.MMFLR.V + 2 Oxidation (M) | 729.3622 |
|  |  |  |  |  |  |  |  |  | K.DSLMLR.C | 734.3998 |
|  |  |  |  |  |  |  |  |  | R.GTGVKALPK.T | 870.5662 |
|  |  |  |  |  |  |  |  |  | K.VHDLMRDIAISK.S + Oxidation (M) | 1413.7764 |
|  |  |  |  |  |  |  |  |  | R.GGAVLQDIKMLTGLR.K | 1571.7950 |
|  |  |  |  |  |  |  |  |  | K.NRSMILPSHESVYSR.K | 1775.8969 |
|  |  |  |  |  |  |  |  |  | K.YHHLDNIWKLLHLK.Y | 1829.8027 |
|  |  |  |  |  |  |  |  |  | K.SANAMAETYFTELKNR.S | 1845.7998 |
|  |  |  |  |  |  |  |  |  | K.FPALMTGVYEEKGAIVPR.G + Oxidation (M) | 1994.0200 |
|  |  |  |  |  |  |  |  |  | R.CLQGARLCATCCLPILR.N | 2062.0957 |
| 14 | metallothionin 3 | *Salvia miltiorrhiza* | 351630004 | 4.56/6.50 | 5.2/13.80 | 23 | 33.33 | 1 | K.CGSCDCADK.S | 1072.0898 |
| 15 | putative Pru du 4.1 allergen, partial | *Prunus dulcis* | 523916666 | 4.79/8.70 | 4.8/13.5 | 68 | 75 | 3 | K.YMVIQGEAGAVIR.G | 1406.8212 |
|  |  |  |  |  |  |  |  |  | K.YMVIQGEAGAVIR.G + Oxidation (M) | 1935.0918 |
|  |  |  |  |  |  |  |  |  | K.DFDQPGTLAPTGLFLGGTK.Y | 1935.0918 |
| 18 | nectarin 5 | *Nicotiana langsdorffii x Nicotiana sanderae* | 30315245 | 5.64/47.75 | 5.5/61 | 94 | 31.71 | 10 | R.GESLDVLRSR.G | 1131.7317 |
|  |  |  |  |  |  |  |  |  | K.SMGEDLFWAIR.G | 1324.7001 |
|  |  |  |  |  |  |  |  |  | K.SMGEDLFWAIR.G + Oxidation (M) | 1340.6962 |
|  |  |  |  |  |  |  |  |  | R.SRGLPPTLYSEAK.A | 1418.7837 |
|  |  |  |  |  |  |  |  |  | R.KSMGEDLFWAIR.G | 1452.8021 |
|  |  |  |  |  |  |  |  |  | R.KSMGEDLFWAIR.G + Oxidation (M) | 1468.8077 |
|  |  |  |  |  |  |  |  |  | K.VIVFTIDKTLEQNATK.L | 1820.0404 |
|  |  |  |  |  |  |  |  |  | R.AAYINYRDLDIGVNNK.K | 1839.0129 |
|  |  |  |  |  |  |  |  |  | R.LLLIMQENFPELGLVR.E | 1884.9784 |
|  |  |  |  |  |  |  |  |  | R.LLLIMQENFPELGLVR.E + Oxidation (M) | 1900.9799 |
| 24 | Glycin-rich RNA binding protein | *Oryza sativa* | 385718874 | 6.31/16.04 | 5.3/16.3 | 46 | 50 | 6 | R.ETGRSR.G | 705.4000 |
|  |  |  |  |  |  |  |  |  | R.RSGGGGGGGYGQR.G | 1165.6093 |
|  |  |  |  |  |  |  |  |  | R.GFGFVTFSSEQAMR.D | 1563.8058 |
|  |  |  |  |  |  |  |  |  | R.GGGGGYGGGGYGGGGGGGGYGQR.R | 1818.9119 |
|  |  |  |  |  |  |  |  |  | R.SRGFGFVTFSSEQAMR.D + Oxidation (M) | 1822.8889 |
|  |  |  |  |  |  |  |  |  | R.EGGYGGGGGYGGGRGGGGYGGGYGSR.G | 2226.1328 |
| 27 | germin-like protein subfamily 1 member 1 precursor | *Arachis hypogaea* | 291042505 | 6.89/24.02 | 5.22/30.67 | 64 | 13.64 | 2 | K.GLNPPHTHPR.A | 1125.6312 |
|  |  |  |  |  |  |  |  |  | R.IDFGPKGLNPPHTHPR.A | 1782.9541 |
| 31 | metallothionin 3 | *Salvia miltiorrhiza* | 351630004 | 4.56/6.50 | 5.7/20.56 | 14 | 33.33 | 1 | K.CGSCDCADK.T | 1072.0820 |
| 34 | dihydrolipoamide dehydrogenase | *Pisum sativum* | 6723874 | 6.69/53.31 | 6.4/56.3 | 71 | 40 | 14 | M.AMANLARR.K | 902.4755 |
|  |  |  |  |  |  |  |  |  | K.VGHVDYDK.V | 932.4987 |
|  |  |  |  |  |  |  |  |  | K.FPFMANSR.A | 969.4814 |
|  |  |  |  |  |  |  |  |  | K.FPFMANSR.A + Oxidation (M) | 985.4707 |
|  |  |  |  |  |  |  |  |  | K.HSFANHGVK.V | 996.5020 |
|  |  |  |  |  |  |  |  |  | K.IIAEKETDK.I | 1046.5435 |
|  |  |  |  |  |  |  |  |  | K.IGVETDKLGR.I | 1087.5292 |
|  |  |  |  |  |  |  |  |  | R.AKAIDNAEGLVK.I | 1228.6318 |
|  |  |  |  |  |  |  |  |  | K.EAAMATYDKPIHI.- | 1459.7557 |
|  |  |  |  |  |  |  |  |  | K.EAAMATYDKPIHI.- + Oxidation (M) | 1475.7474 |
|  |  |  |  |  |  |  |  |  | R.VCHAHPTMSEAIK.E | 1480.7311 |
|  |  |  |  |  |  |  |  |  | K.ALLHSSHMYHEAK.H | 1523.7671 |
|  |  |  |  |  |  |  |  |  | K.ALLHSSHMYHEAK.H + Oxidation (M) | 1539.7733 |
|  |  |  |  |  |  |  |  |  | K.AEEDGVACVEYLAGK.V | 1610.7794 |
| 36 | retrotransposon protein, putative, Ty3-gypsy subclass | *Oryza sativa Japonica Group* | 108708895 | 8.31/172.77 | 5.5/85.5 | 50 | 13.55 | 19 | R.HVYRK.D | 702.3615 |
|  |  |  |  |  |  |  |  |  | K.AIENMK.S | 705.3985 |
|  |  |  |  |  |  |  |  |  | K.SCLSDK.N | 709.3384 |
|  |  |  |  |  |  |  |  |  | R.DHLNGR.R | 711.3475 |
|  |  |  |  |  |  |  |  |  | R.MTSETK.N + Oxidation (M) | 712.3179 |
|  |  |  |  |  |  |  |  |  | R.NIKFAR.V | 748.3657 |
|  |  |  |  |  |  |  |  |  | K.VFLSAMK.T + Oxidation (M) | 811.3972 |
|  |  |  |  |  |  |  |  |  | R.TFKSGFR.D | 842.5206 |
|  |  |  |  |  |  |  |  |  | R.AAAALGVKR.L | 856.5327 |
|  |  |  |  |  |  |  |  |  | R.RFDECR.N | 882.4579 |
|  |  |  |  |  |  |  |  |  | R.FVARMGER.G + Oxidation (M) | 981.5145 |
|  |  |  |  |  |  |  |  |  | K.LQRFAPDR.K | 1002.5486 |
|  |  |  |  |  |  |  |  |  | R.ETGIEANPEK.I | 1087.5548 |
|  |  |  |  |  |  |  |  |  | R.VTFSQANNFR.T | 1183.5590 |
|  |  |  |  |  |  |  |  |  | R.ESPHRATPSPR.G | 1234.6099 |
|  |  |  |  |  |  |  |  |  | R.NTIPEITDASVIR.T | 1428.7703 |
|  |  |  |  |  |  |  |  |  | R.MGERGQPFFALLK.K + Oxidation (M) | 1509.7837 |
|  |  |  |  |  |  |  |  |  | R.DEDKEQGATLISDR.F | 1576.7213 |
|  |  |  |  |  |  |  |  |  | R.TEQITFDVAEFDTTYNAIIGRTALAR.F | 2916.3264 |
| 39 | 1-aminocyclopropane-1-carboxylate oxidase | *Musa ABB Group* | 134260548 | 5.25/35.87 | 4.67/23.67 | 64 | 41.94 | 11 | K.GPTFGTK.V | 707.3486 |
|  |  |  |  |  |  |  |  |  | K.AFSNGSK.G | 710.3600 |
|  |  |  |  |  |  |  |  |  | K.LYVGHK.F | 716.3436 |
|  |  |  |  |  |  |  |  |  | K.YREQK.F | 723.3677 |
|  |  |  |  |  |  |  |  |  | K.EAEEKK.E | 733.3299 |
|  |  |  |  |  |  |  |  |  | K.DGEWLDVPPVR.H | 1282.6838 |
|  |  |  |  |  |  |  |  |  | K.LSGGERGAAMEILR.D + Oxidation (M) | 1475.7352 |
|  |  |  |  |  |  |  |  |  | K.EVYPRFVFEDYMK.L + Oxidation (M) | 1738.8097 |
|  |  |  |  |  |  |  |  |  | K.VSSYPPCPRPDLVKGLR.A | 1940.9205 |
|  |  |  |  |  |  |  |  |  | R.HAIVVNLGDQLEVITNGKYK.S | 2211.0891 |
|  |  |  |  |  |  |  |  |  | R.HLPVSNISEIPDLDDQYRK.A | 2239.1167 |
| 44 | Peroxisomal membrane protein PMP22 | *Aegilops tauschii* | 475601557 | 9.94/46.15 | 6.4/29.5 | 52 | 28.57 | 10 | K.VLDYIFKGK.K | 1082.4937 |
|  |  |  |  |  |  |  |  |  | R.MAILPMGTGTR.G + 2 Oxidation (M) | 1179.5980 |
|  |  |  |  |  |  |  |  |  | K.GQLGAARTHDTR.G | 1282.6832 |
|  |  |  |  |  |  |  |  |  | R.QSHVFEAVIDR.F | 1300.5466 |
|  |  |  |  |  |  |  |  |  | K.CAGGESGSMASYR.S | 1332.7490 |
|  |  |  |  |  |  |  |  |  | M.AGGTGGAGGGTGGGDSLAR.R | 1475.7485 |
|  |  |  |  |  |  |  |  |  | K.MITAGCLAGVSDSVAQK.L | 1707.7795 |
|  |  |  |  |  |  |  |  |  | K.CAGGESGSMASYRSAVAR.L + Oxidation (M) | 1832.8414 |
|  |  |  |  |  |  |  |  |  | K.ESSSAMSVTVGVDERER.G | 1838.9152 |
|  |  |  |  |  |  |  |  |  | K.MITAGCLAGVSDSVAQKLSGYQK.I | 2383.9272 |
| 50 | pathogenesis related protein PR10 | *Prunus persica* | 159794683 | 5.79/17.64 | 5.89/17.98 | 311 | 93.75 | 8 | K.STSHYHTK.G | 960.5273 |
|  |  |  |  |  |  |  |  |  | K.GDVEIKEEHVK.A | 1282.6847 |
|  |  |  |  |  |  |  |  |  | K.AFVLDADNLVPK.I | 1301.7216 |
|  |  |  |  |  |  |  |  |  | K.ITFGEGSQYGYVK.H | 1448.7175 |
|  |  |  |  |  |  |  |  |  | K.HSEILEGDGGPGTIK.K | 1509.7731 |
|  |  |  |  |  |  |  |  |  | K.KITFGEGSQYGYVK.H | 1576.8143 |
|  |  |  |  |  |  |  |  |  | K.HSEILEGDGGPGTIKK.I | 1637.8698 |
|  |  |  |  |  |  |  |  |  | M.GVFTYESEFTSEIPPPR.L | 1955.9598 |
| 51 | major cherry allergen Pru av 1.0201 | *Prunus avium* | 44409451 | 4.98/17.35 | 5.31/35 | 384 | 75 | 8 | K.HRIDGLDK.D | 953.5085 |
|  |  |  |  |  |  |  |  |  | K.LVASADGGSIIK.S | 1130.6309 |
|  |  |  |  |  |  |  |  |  | K.ALVLEADTLIPK.I | 1282.7422 |
|  |  |  |  |  |  |  |  |  | K.ISFGEGSHYSYVK.H | 1473.6865 |
|  |  |  |  |  |  |  |  |  | K.LIENYLAANPDACN.- | 1577.7186 |
|  |  |  |  |  |  |  |  |  | K.KISFGEGSHYSYVK.H | 1601.7809 |
|  |  |  |  |  |  |  |  |  | M.GVFTYSDESTSVIPPPR.L | 1851.9209 |
|  |  |  |  |  |  |  |  |  | K.DNFVYNYTLVEGDALSDK.I | 2062.9575 |
| 53 | thioredoxin h-type | *Vitis vinifera* | 452114374 | 5.15/14.18 | 5.5/17.57 | 42 | 58.33 | 7 | R.SSRSHK.M | 701.3367 |
|  |  |  |  |  |  |  |  |  | K.ESRVVK.V | 717.2985 |
|  |  |  |  |  |  |  |  |  | R.TEAFIR.S | 736.3143 |
|  |  |  |  |  |  |  |  |  | R.EGAQVDK.L | 746.3192 |
|  |  |  |  |  |  |  |  |  | M.AGNQQLK.E | 758.3231 |
|  |  |  |  |  |  |  |  |  | -.MAGNQQLK.E + Oxidation (M) | 905.4149 |
|  |  |  |  |  |  |  |  |  | -.MAGNQQLKESR.V + Oxidation (M) | 1277.6254 |
| 55 | metallothionin 3 | *Salvia miltiorrhiza* | 351630004 | 4.56/6.50 | 5.1/49.2 | 16 | 33.33 | 1 | K.CGSCDCADK.T | 1072.0980 |
| 63 | ascorbate peroxidase | *Vigna luteola* | 187962068 | 5.14/23.87 | 6.3/36 | 172 | 31.82 | 5 | K.TGGPFGTMK.H + Oxidation (M) | 911.4178 |
|  |  |  |  |  |  |  |  |  | K.RCAPLMLR.L | 1016.4351 |
|  |  |  |  |  |  |  |  |  | R.EDKPEPPPEGR.L | 1250.5796 |
|  |  |  |  |  |  |  |  |  | K.HPAELAHGANNGLDIAVR.L | 1854.8523 |
|  |  |  |  |  |  |  |  |  | K.YAADEDAFFADYAVAHQK.L | 2031.8558 |
| 68 | thaumatin-like protein 2 | *Prunus persica* | 359744030 | 4.83/25.78 | 5.32/36 | 98 | 20.83 | 3 | K.DQCPQAYSYAYDDK.S | 1723.6611 |
|  |  |  |  |  |  |  |  |  | K.IFKDQCPQAYSYAYDDK.S | 2111.9358 |
|  |  |  |  |  |  |  |  |  | K.SSTFTCSARPDYLITFCP.- | 2122.9316 |
| 60 | resistance protein | *Arachis cardenasii* | 37222029 | 9.51/25.39 | 5.38/45.2 | 32 | 18.18 | 3 | K.SNVEIPRQAK.K | 1141.6318 |
|  |  |  |  |  |  |  |  |  | K.DYDNAKVMNDIR.N | 1453.7664 |
|  |  |  |  |  |  |  |  |  | K.LELQEVGVPNPMK.A | 1453.7664 |
| 77 | PREDICTED: brefeldin A resistance protein-like | *Solanum lycopersicum* | 460379917 | 4.86/47.69 | 4.91/65.34 | 74 | 27.27 | 10 | K.VNVSTTGEGK.G | 991.4760 |
|  |  |  |  |  |  |  |  |  | K.ASEEVMASR.R + Oxidation (M) | 995.4915 |
|  |  |  |  |  |  |  |  |  | K.DASIVEDFR.A | 1051.4850 |
|  |  |  |  |  |  |  |  |  | K.GLSTIALKFK.D | 1077.5128 |
|  |  |  |  |  |  |  |  |  | K.ASEEVMASRR.I + Oxidation (M) | 1151.5642 |
|  |  |  |  |  |  |  |  |  | K.FKDASIVEDFR.A | 1326.6342 |
|  |  |  |  |  |  |  |  |  | K.ETNDVCKEELK.E | 1364.6423 |
|  |  |  |  |  |  |  |  |  | -.MGDAENSLQPSKK.R + Oxidation (M) | 1420.7163 |
|  |  |  |  |  |  |  |  |  | K.VETPNEADKPESAEEK.V | 1772.8549 |
|  |  |  |  |  |  |  |  |  | R.DNPGLDDDENEAELETGSFKK.A | 2322.9568 |
| 73 | Catalase isozyme 2 | *Triticum urartu* | 473958051 | 6.71/58.79 | 5.93/21.67 | 58 | 33.33 | 12 | -.MEGEAR.T + Oxidation (M) | 708.3010 |
|  |  |  |  |  |  |  |  |  | K.VSHELR.V | 740.3067 |
|  |  |  |  |  |  |  |  |  | R.LNVKPSM.- + Oxidation (M) | 804.2504 |
|  |  |  |  |  |  |  |  |  | K.SHVQEYWR.V | 1104.5533 |
|  |  |  |  |  |  |  |  |  | R.SWAPDRQDR.F | 1130.5065 |
|  |  |  |  |  |  |  |  |  | K.KENDFVQPGER.Y | 1318.6459 |
|  |  |  |  |  |  |  |  |  | K.NNHYDGAMNFMHR.D | 1606.7965 |
|  |  |  |  |  |  |  |  |  | K.TWPEDLVPLQPVGR.L | 1606.7965 |
|  |  |  |  |  |  |  |  |  | R.FSTVIHERGSPETIR.D | 1728.8293 |
|  |  |  |  |  |  |  |  |  | R.HMDGFGVNTYTFVSR.A + Oxidation (M) | 1746.8341 |
|  |  |  |  |  |  |  |  |  | K.MLQCRVFAYADTQR.Y | 1758.8293 |
|  |  |  |  |  |  |  |  |  | K.NNHYDGAMNFMHRDEEVDYYPSR.H | 2860.1868 |
| 88 | Catalase isozyme 2 | *Triticum urartu* | 473958051 | 6.71/58.79 | 4.83/30.4 | 58 | 33.33 | 12 | -.MEGEAR.T + Oxidation (M) | 708.3010 |
|  |  |  |  |  |  |  |  |  | K.VSHELR.V | 740.3067 |
|  |  |  |  |  |  |  |  |  | R.LNVKPSM.- + Oxidation (M) | 804.2504 |
|  |  |  |  |  |  |  |  |  | K.SHVQEYWR.V | 1104.5533 |
|  |  |  |  |  |  |  |  |  | R.SWAPDRQDR.F | 1130.5065 |
|  |  |  |  |  |  |  |  |  | K.KENDFVQPGER.Y | 1318.6459 |
|  |  |  |  |  |  |  |  |  | K.NNHYDGAMNFMHR.D | 1606.7965 |
|  |  |  |  |  |  |  |  |  | K.TWPEDLVPLQPVGR.L | 1606.7965 |
|  |  |  |  |  |  |  |  |  | R.FSTVIHERGSPETIR.D | 1728.8293 |
|  |  |  |  |  |  |  |  |  | R.HMDGFGVNTYTFVSR.A + Oxidation (M) | 1746.8341 |
|  |  |  |  |  |  |  |  |  | K.MLQCRVFAYADTQR.Y | 1758.8293 |
|  |  |  |  |  |  |  |  |  | K.NNHYDGAMNFMHRDEEVDYYPSR.H | 2860.1868 |
| 86 | senescence-associated protein 6 | *Hemerocallis hybrid cultivar* | 3551956 | 5.7/34.11 | 5.5/39.7 | 63 | 20.69 | 3 | K.SNLHHVWDR.E | 1163.6299 |
|  |  |  |  |  |  |  |  |  | K.MGYSCVVLGLILVSLPGAWPWSK.E | 2533.4004 |
|  |  |  |  |  |  |  |  |  | K.GAEDMCVAGAVHNYTTQLMHYR.D + 2 Oxidation (M) | 2556.2803 |
| **Molecular chaperones** | | | | | | | | | | |
| 30 | Chaperone protein dnaJ | *Medicago truncatula* | 355505325 | 9.79/12.34 | 5.69/21.3 | 54 | 63.64 | 5 | K.QNKMNK.V + Oxidation (M) | 778.3832 |
|  |  |  |  |  |  |  |  |  | M.AATTAVGVIGGNGSSWMQFGR.K + Oxidation (M) | 2082.9983 |
|  |  |  |  |  |  |  |  |  | M.AATTAVGVIGGNGSSWMQFGRK.E + Oxidation (M) | 2211.0803 |
|  |  |  |  |  |  |  |  |  | R.DCGVQFHLINEAYDVSFPK.I | 2239.1196 |
|  |  |  |  |  |  |  |  |  | -.MAATTAVGVIGGNGSSWMQFGRK.E | 2326.2119 |
| 32 | cytosolic class I small heat shock protein type 1 | *Rhododendron formosanum* | 283482294 | 5.35/16.25 | 5.55/21 | 92 | 57.14 | 4 | R.ILQISGER.S | 915.5457 |
|  |  |  |  |  |  |  |  |  | R.FQLPENAK.M | 946.5167 |
|  |  |  |  |  |  |  |  |  | K.ETPEAHVFK.A | 1057.5536 |
|  |  |  |  |  |  |  |  |  | R.RFQLPENAK.M | 1102.6245 |
| 75 | small heat shock protein | *Prunus persica* | 41059801 | 5.98/17.39 | 6.03/17.54 | 149 | 46.67 | 5 | R.SREQEEK.N | 905.4476 |
|  |  |  |  |  |  |  |  |  | R.FRLPDNAK.I | 960.5172 |
|  |  |  |  |  |  |  |  |  | M.ALSLFGGRR.S | 976.5095 |
|  |  |  |  |  |  |  |  |  | R.ETTAIANTR.I | 976.5095 |
|  |  |  |  |  |  |  |  |  | K.ETPEAHIFIADLPGLK.K | 1750.8953 |
| 78 | Stromal 70 kDa heat shock-related family protein | *Populus trichocarpa* | 566259926 | 5.24/75.34 | 5.15/66.78 | 583 | 37.14 | 21 | R.DEGIDLLK.D | 902.4775 |
|  |  |  |  |  |  |  |  |  | R.LDGIPPAPR.G | 935.5110 |
|  |  |  |  |  |  |  |  |  | R.RYSTGPVR.V | 935.5110 |
|  |  |  |  |  |  |  |  |  | K.HIETTLTR.A | 970.5121 |
|  |  |  |  |  |  |  |  |  | R.VVRDENGNVK.L | 1129.5709 |
|  |  |  |  |  |  |  |  |  | K.HIETTLTRAK.F | 1169.6045 |
|  |  |  |  |  |  |  |  |  | K.DAIAGGSTQVMK.D | 1177.5938 |
|  |  |  |  |  |  |  |  |  | R.KMSEVDEESK.Q + Oxidation (M) | 1197.6334 |
|  |  |  |  |  |  |  |  |  | K.DLDEVILVGGSTR.I | 1373.6913 |
|  |  |  |  |  |  |  |  |  | K.NQADSVVYQTEK.Q | 1381.6255 |
|  |  |  |  |  |  |  |  |  | K.FEELCSDLLDR.L | 1396.6180 |
|  |  |  |  |  |  |  |  |  | K.QFAAEEISAQVLR.K | 1461.7289 |
|  |  |  |  |  |  |  |  |  | K.GPDGDVIDADFTDSK.- | 1551.6682 |
|  |  |  |  |  |  |  |  |  | K.AVVTVPAYFNDSQR.T | 1566.7513 |
|  |  |  |  |  |  |  |  |  | R.QAVVNPENTFFSVK.R | 1579.7670 |
|  |  |  |  |  |  |  |  |  | R.GTTATSLKHTNNTSR.R | 1588.7369 |
|  |  |  |  |  |  |  |  |  | R.AKFEELCSDLLDR.L | 1595.7258 |
|  |  |  |  |  |  |  |  |  | R.QAVVNPENTFFSVKR.F | 1735.8668 |
|  |  |  |  |  |  |  |  |  | K.QDITITGASTLPSDEVER.M | 1931.9082 |
|  |  |  |  |  |  |  |  |  | K.KQDITITGASTLPSDEVER.M | 2060.0022 |
|  |  |  |  |  |  |  |  |  | K.SEVFSTAADGQTSVEINVLQGER.E | 2437.1377 |
| 80 | endoplasmin, putative | *Ricinus communis* | 223551251 | 4.86/93.31 | 5.1/86.98 | 256 | 27.16 | 15 | K.QHVWESK.A | 913.4894 |
|  |  |  |  |  |  |  |  |  | K.SGTSAFVEK.M | 925.5093 |
|  |  |  |  |  |  |  |  |  | K.FWNEFGK.S | 927.4767 |
|  |  |  |  |  |  |  |  |  | K.YGWSANMER.I | 1113.5330 |
|  |  |  |  |  |  |  |  |  | K.YGWSANMER.I + Oxidation (M) | 1129.5278 |
|  |  |  |  |  |  |  |  |  | K.LTSLDQYISR.M | 1195.6890 |
|  |  |  |  |  |  |  |  |  | R.EMLQQHSSLK.T | 1200.6759 |
|  |  |  |  |  |  |  |  |  | R.EMLQQHSSLK.T + Oxidation (M) | 1216.6799 |
|  |  |  |  |  |  |  |  |  | K.GLVDSDTLPLNVSR.E | 1485.8658 |
|  |  |  |  |  |  |  |  |  | K.HNDDKQHVWESK.A | 1522.7887 |
|  |  |  |  |  |  |  |  |  | R.ELISNASDALDKIR.F | 1544.8938 |
|  |  |  |  |  |  |  |  |  | R.VFISDEFDELLPK.Y | 1551.8679 |
|  |  |  |  |  |  |  |  |  | K.AESSSSDEEEETEK.G | 1556.7798 |
|  |  |  |  |  |  |  |  |  | K.APHDLYESYYNANK.S | 1684.8530 |
|  |  |  |  |  |  |  |  |  | R.RVFISDEFDELLPK.Y | 1707.9729 |
| 81 | heat shock cognate protein 70 | *Eutrema halophilum* | 41584275 | 5.04/71.25 | 5.3/70.5 | 310 | 44.62 | 23 | K.FELSGIPPAPR.G | 1183.7096 |
|  |  |  |  |  |  |  |  |  | K.DAGVIAGLNVMR.I | 1215.7161 |
|  |  |  |  |  |  |  |  |  | R.VEIIANDQGNR.T | 1228.6923 |
|  |  |  |  |  |  |  |  |  | R.MVNHFVQEFK.R | 1278.6981 |
|  |  |  |  |  |  |  |  |  | R.EIAEAYLGVSIK.N | 1292.7162 |
|  |  |  |  |  |  |  |  |  | R.MVNHFVQEFK.R + Oxidation (M) | 1294.6967 |
|  |  |  |  |  |  |  |  |  | R.GQEPRTTTLLGK.F | 1300.6906 |
|  |  |  |  |  |  |  |  |  | R.FEELNMDLFR.K | 1313.6906 |
|  |  |  |  |  |  |  |  |  | R.FEELNMDLFR.K + Oxidation (M) | 1329.6869 |
|  |  |  |  |  |  |  |  |  | K.NALENYAYNMR.N | 1358.6879 |
|  |  |  |  |  |  |  |  |  | K.NALENYAYNMR.N + Oxidation (M) | 1374.6835 |
|  |  |  |  |  |  |  |  |  | K.ELESICNPIIAK.I | 1386.7933 |
|  |  |  |  |  |  |  |  |  | K.STVHDVVLVGGSTR.I | 1426.8389 |
|  |  |  |  |  |  |  |  |  | K.VQQLLQDFFNGK.E | 1436.8274 |
|  |  |  |  |  |  |  |  |  | R.TTPSYVAFTDSER.L | 1473.7639 |
|  |  |  |  |  |  |  |  |  | R.ARFEELNMDLFR.K | 1540.8412 |
|  |  |  |  |  |  |  |  |  | R.ARFEELNMDLFR.K + Oxidation (M) | 1556.8362 |
|  |  |  |  |  |  |  |  |  | K.MKELESICNPIIAK.I | 1645.9496 |
|  |  |  |  |  |  |  |  |  | R.IINEPTAAAIAYGLDK.K | 1659.9884 |
|  |  |  |  |  |  |  |  |  | K.MKELESICNPIIAK.I + Oxidation (M) | 1661.9570 |
|  |  |  |  |  |  |  |  |  | K.ATAGDTHLGGEDFDNR.M | 1675.8259 |
|  |  |  |  |  |  |  |  |  | K.NAVVTVPAYFNDSQR.Q | 1680.9264 |
|  |  |  |  |  |  |  |  |  | R.IINEPTAAAIAYGLDKK.A | 1788.0869 |
| 82 | Stromal 70 kDa heat shock-related family protein | *Populus trichocarpa* | 550312340 | 5.24/75.34 | 5.48/61.5 | 583 | 37.14 | 21 | R.DEGIDLLK.D | 902.4775 |
|  |  |  |  |  |  |  |  |  | R.LDGIPPAPR.G | 935.5110 |
|  |  |  |  |  |  |  |  |  | R.RYSTGPVR.V | 935.5110 |
|  |  |  |  |  |  |  |  |  | K.HIETTLTR.A | 970.5121 |
|  |  |  |  |  |  |  |  |  | R.VVRDENGNVK.L | 1129.5709 |
|  |  |  |  |  |  |  |  |  | K.HIETTLTRAK.F | 1169.6045 |
|  |  |  |  |  |  |  |  |  | K.DAIAGGSTQVMK.D | 1177.5938 |
|  |  |  |  |  |  |  |  |  | R.KMSEVDEESK.Q + Oxidation (M) | 1197.6334 |
|  |  |  |  |  |  |  |  |  | K.DLDEVILVGGSTR.I | 1373.6913 |
|  |  |  |  |  |  |  |  |  | K.NQADSVVYQTEK.Q | 1381.6255 |
|  |  |  |  |  |  |  |  |  | K.FEELCSDLLDR.L | 1396.6180 |
|  |  |  |  |  |  |  |  |  | K.QFAAEEISAQVLR.K | 1461.7289 |
|  |  |  |  |  |  |  |  |  | K.GPDGDVIDADFTDSK.- | 1551.6682 |
|  |  |  |  |  |  |  |  |  | K.AVVTVPAYFNDSQR.T | 1566.7513 |
|  |  |  |  |  |  |  |  |  | R.QAVVNPENTFFSVK.R | 1579.7670 |
|  |  |  |  |  |  |  |  |  | R.GTTATSLKHTNNTSR.R | 1588.7369 |
|  |  |  |  |  |  |  |  |  | R.AKFEELCSDLLDR.L | 1595.7258 |
|  |  |  |  |  |  |  |  |  | R.QAVVNPENTFFSVKR.F | 1735.8668 |
|  |  |  |  |  |  |  |  |  | K.QDITITGASTLPSDEVER.M | 1931.9082 |
|  |  |  |  |  |  |  |  |  | K.KQDITITGASTLPSDEVER.M | 2060.0022 |
|  |  |  |  |  |  |  |  |  | K.SEVFSTAADGQTSVEINVLQGER.E | 2437.1377 |
| 94 | copper chaperone | *Jatropha curcas* | 257219554 | 4.93/9.85 | 5.13/18.1 | 60 | 55.56 | 3 | K.VGMSCQGCVGAVK.R | 1352.5513 |
|  |  |  |  |  |  |  |  |  | K.VGMSCQGCVGAVK.R + Oxidation (M) | 1368.5422 |
|  |  |  |  |  |  |  |  |  | K.MEGVESYDIDLQEQKVTVK.G | 2211.0044 |
| 96 | Luminal-binding protein 5 | *Aegilops tauschii* | 475534891 | 4.96/54.20 | 5.54/13.5 | 52 | 18.37 | 5 | K.FDLSGIPPAPR.G | 1169.4803 |
|  |  |  |  |  |  |  |  |  | K.KTQVFTTYQDK.Q | 1358.5786 |
|  |  |  |  |  |  |  |  |  | R.HSQEEIDRMVR.E + Oxidation (M) | 1415.6138 |
|  |  |  |  |  |  |  |  |  | R.LLGKFDLSGIPPAPR.G | 1580.6886 |
|  |  |  |  |  |  |  |  |  | R.MVREAEEFMEEDR.K | 1670.8243 |
| **Protein degradation** | | | | | | | | | | |
| 16 | PREDICTED: putative DNA repair protein RAD23-3-like | *Fragaria vesca subsp. Vesca* | 470126218 | 4.63/42.14 | 4.8/48 | 109 | 20 | 4 | R.LEAMGFDR.A | 938.4293 |
|  |  |  |  |  |  |  |  |  | R.LEAMGFDR.A + Oxidation (M) | 954.4243 |
|  |  |  |  |  |  |  |  |  | R.LIQQHQADFLR.L | 1368.7155 |
|  |  |  |  |  |  |  |  |  | R.ALVLEVFVACNR.N | 1390.6821 |
| 25 | RAD23 protein | *Solanum lycopersicum var. cerasiforme* | 5640111 | 4.65/41.51 | 4.59/46.78 | 106 | 26.32 | 7 | R.AAYNNPER.A | 934.4854 |
|  |  |  |  |  |  |  |  |  | R.LEAMGFDR.A | 938.4799 |
|  |  |  |  |  |  |  |  |  | R.LEAMGFDR.A + Oxidation (M) | 954.4797 |
|  |  |  |  |  |  |  |  |  | R.NSPQFQALR.A | 1060.5990 |
|  |  |  |  |  |  |  |  |  | K.VLKDTTTLEENK.V | 1390.7744 |
|  |  |  |  |  |  |  |  |  | R.ALVLEVYFACNK.N | 1426.7939 |
|  |  |  |  |  |  |  |  |  | K.VAENSFVVIMLSK.N | 1436.7959 |
| 72 | proteasome subunit alpha type-5 | *Glycine max* | 351721357 | 4.7/25.98 | 4.83/30.5 | 116 | 43.48 | 8 | R.TLVEHAR.V | 825.4356 |
|  |  |  |  |  |  |  |  |  | R.VETQNHR.F | 883.4166 |
|  |  |  |  |  |  |  |  |  | R.GVNTFSPEGR.L | 1063.4929 |
|  |  |  |  |  |  |  |  |  | R.ITSPLLEPSSVEK.I | 1399.7252 |
|  |  |  |  |  |  |  |  |  | R.LFQVEYAIEAIK.L | 1423.7318 |
|  |  |  |  |  |  |  |  |  | K.VAPTYHLYTPSEVEAVISR.L | 2132.0378 |
|  |  |  |  |  |  |  |  |  | R.FSYGEPMTVESTTQALCDLALR.F | 2489.0923 |
|  |  |  |  |  |  |  |  |  | R.FSYGEPMTVESTTQALCDLALR.F + Oxidation (M) | 2505.0928 |
| **Signal transduction** | | | | | | | | | | |
| 21 | Glycogen synthase kinase-3 beta, putative | *Ricinus communis* | 223545698 | 8.59/46.22 | 5.34/44.69 | 55 | 32.5 | 11 | R.VIKHYNK.M | 901.5191 |
|  |  |  |  |  |  |  |  |  | K.HYNKMGQR.M + Oxidation (M) | 1049.5171 |
|  |  |  |  |  |  |  |  |  | R.MPPEAVDLVSR.L + Oxidation (M) | 1229.7025 |
|  |  |  |  |  |  |  |  |  | -.MASVSVAPTSGIR.D | 1275.6681 |
|  |  |  |  |  |  |  |  |  | K.GEPNISYICSR.Y | 1295.6251 |
|  |  |  |  |  |  |  |  |  | K.CMNPNYTEFK.F | 1303.6510 |
|  |  |  |  |  |  |  |  |  | R.LPDEMNDMKIR.D + 2 Oxidation (M) | 1393.7013 |
|  |  |  |  |  |  |  |  |  | K.GVPVEVLVKLIPEHAR.K | 1755.8528 |
|  |  |  |  |  |  |  |  |  | R.EEIKCMNPNYTEFK.F | 1802.9539 |
|  |  |  |  |  |  |  |  |  | R.ELQTMRLLDHPNVVSLK.H + Oxidation (M) | 2008.8951 |
|  |  |  |  |  |  |  |  |  | R.STALDALIHPFFDELRDPNTR.L | 2428.2539 |
| 33 | Calcium-binding EF-hand family protein | *Theobroma cacao* | 508777222 | 4.82/16.51 | 4.38/14.76 | 185 | 92.86 | 8 | R.HILTSIGEK.L | 997.5875 |
|  |  |  |  |  |  |  |  |  | K.LTAPFDFPR.F | 1063.5769 |
|  |  |  |  |  |  |  |  |  | K.HMKPEPFDR.Q | 1156.6025 |
|  |  |  |  |  |  |  |  |  | K.HMKPEPFDR.Q + Oxidation (M) | 1172.5715 |
|  |  |  |  |  |  |  |  |  | K.IAPSELGILMR.S | 1199.7023 |
|  |  |  |  |  |  |  |  |  | R.SLGGNPTQAQLK.A | 1213.6730 |
|  |  |  |  |  |  |  |  |  | K.IAPSELGILMR.S + Oxidation (M) | 1215.6921 |
|  |  |  |  |  |  |  |  |  | K.LEPSEFDEWIR.E | 1420.7024 |
| 41 | Transducin/WD40 repeat-like superfamily protein, putative | *Theobroma cacao* | 508785877 | 6.03/18.71 | 5.2/14.36 | 53 | 25 | 2 | R.IIPFADEKK.I | 1060.5278 |
|  |  |  |  |  |  |  |  |  | R.LGEILENGQVR.T | 1227.7126 |
| 43 | ethylene receptor-like protein precursor | *Solanum lycopersicum* | 350534618 | 7.28/85.05 | 4.4/18.43 | 55 | 20 | 13 | K.AREFMLR.R | 922.4406 |
|  |  |  |  |  |  |  |  |  | K.AREFMLR.R + Oxidation (M) | 938.5546 |
|  |  |  |  |  |  |  |  |  | K.VLLHANRGMP.- | 1107.5664 |
|  |  |  |  |  |  |  |  |  | K.GYNITIEVEK.S | 1165.6100 |
|  |  |  |  |  |  |  |  |  | R.SSFQTVMSHR.L | 1179.6226 |
|  |  |  |  |  |  |  |  |  | K.ENVKFPLEMK.H | 1234.6919 |
|  |  |  |  |  |  |  |  |  | R.DEQQLLVHSIIK.S | 1422.7709 |
|  |  |  |  |  |  |  |  |  | R.FWLNQEVEIVR.A | 1432.7334 |
|  |  |  |  |  |  |  |  |  | R.GVEVLLADYDDSNR.A | 1565.7732 |
|  |  |  |  |  |  |  |  |  | K.GYNITIEVEKSFPNK.V | 1738.8450 |
|  |  |  |  |  |  |  |  |  | K.SIDRHTILYTTLVGLSK.L | 1916.9935 |
|  |  |  |  |  |  |  |  |  | R.RPMHSILGLLSMLQEQK.L + 2 Oxidation (M) | 2012.9868 |
|  |  |  |  |  |  |  |  |  | R.QTWPLIIGLAAITDEDIRK.C | 2153.0686 |
| 62 | tyrosine-specific protein phosphatase-like protein | *Arabidopsis thaliana* | 9758683 | 9.37/4.99 | 6.78/41.3 | 54 | 19.05 | 4 | R.KMLDLVLLK.T | 1072.6667 |
|  |  |  |  |  |  |  |  |  | R.EELKNWQR.V | 1102.6499 |
|  |  |  |  |  |  |  |  |  | K.LLLLSKHGVLR.V | 1248.5548 |
|  |  |  |  |  |  |  |  |  | K.RVVAVIHELLSLTVEK.M | 1806.0547 |
| 74 | calcineurin B-like | *Oryza sativa Japonica Group* | 55773930 | 12.07/31.45 | 5.12/19 | 54 | 46.43 | 11 | R.RPTATATAK.E | 916.4833 |
|  |  |  |  |  |  |  |  |  | -.MTSPTTAAAR.R | 1006.5188 |
|  |  |  |  |  |  |  |  |  | K.GNGGGAKLGFR.A | 1033.4949 |
|  |  |  |  |  |  |  |  |  | R.TGPDWAEER.E | 1060.5029 |
|  |  |  |  |  |  |  |  |  | K.LGFRAAAAEGR.R | 1118.4821 |
|  |  |  |  |  |  |  |  |  | R.LTGGTHLSATAR.S | 1184.5725 |
|  |  |  |  |  |  |  |  |  | R.RPTATATAKER.S | 1201.6014 |
|  |  |  |  |  |  |  |  |  | R.AATGRGDTGGPFK.G | 1234.6495 |
|  |  |  |  |  |  |  |  |  | R.LRTGPDWAEER.E | 1329.6125 |
|  |  |  |  |  |  |  |  |  | R.RLTGGTHLSATAR.S | 1340.6566 |
|  |  |  |  |  |  |  |  |  | R.LTGGTHLSATARSR.G | 1427.7524 |
| 93 | TCTP protein | *Fragaria x ananassa* | 1922278 | 4.37/19.04 | 4.89/22.3 | 186 | 47.06 | 5 | K.QFVTWVK.R | 907.4704 |
|  |  |  |  |  |  |  |  |  | K.KQFVTWVK.R | 1035.5603 |
|  |  |  |  |  |  |  |  |  | K.VVDIVDTFR.L | 1063.5393 |
|  |  |  |  |  |  |  |  |  | R.LQEQPPFDK.K | 1101.5133 |
|  |  |  |  |  |  |  |  |  | R.LQEQPPFDKK.Q | 1229.5988 |
| **Other Materials Metabolism** | | | | | | | | | | |
| 7 | O-acetylserine(thiol)-lyase | *Sesamum indicum* | 158263556 | 5.62/34.33 | 6.1/34.6 | 122 | 37.5 | 8 | R.YLSSVLFESVR.R | 1299.7632 |
|  |  |  |  |  |  |  |  |  | R.AFGAELVLTDPAK.G | 1331.7828 |
|  |  |  |  |  |  |  |  |  | K.LIVVIFPSFGER.Y | 1376.8674 |
|  |  |  |  |  |  |  |  |  | K.LIITMPSSMSLER.R | 1477.8518 |
|  |  |  |  |  |  |  |  |  | K.LIITMPSSMSLER.R + Oxidation (M) | 1493.8397 |
|  |  |  |  |  |  |  |  |  | K.EGLLVGISSGAAAAAAIR.I | 1627.0074 |
|  |  |  |  |  |  |  |  |  | K.IDAFVSGIGTGGTITGAGK.F | 1721.9978 |
|  |  |  |  |  |  |  |  |  | K.LYGVEPVESPILSGGKPGPHK.I | 2161.3020 |
| 65 | caleosin CLO1-6 | *Brassica napus* | 196122126 | 5.81/28.14 | 6.2/22.56 | 39 | 25 | 3 | M.STATEIMER.D + Oxidation (M) | 1053.2449 |
|  |  |  |  |  |  |  |  |  | R.DAMATVAPYAPVTFHR.R | 1746.8671 |
|  |  |  |  |  |  |  |  |  | R.LPKPYMPRALQAPDR.E + Oxidation (M) | 1768.8597 |
| 70 | isopentenyl diphosphate isomerase | *Solanum lycopersicum* | 350538405 | 5.06/27.19 | 5.14/30.45 | 139 | 39.13 | 6 | K.YVNQEQLK.E | 1021.5245 |
|  |  |  |  |  |  |  |  |  | R.LVVDNFLFK.W | 1094.6140 |
|  |  |  |  |  |  |  |  |  | K.IESENLLHR.A | 1110.5831 |
|  |  |  |  |  |  |  |  |  | R.AFSVFLFNSK.Y | 1159.6010 |
|  |  |  |  |  |  |  |  |  | R.DVNVHPNPDEVADIK.Y | 1661.8033 |
|  |  |  |  |  |  |  |  |  | K.VTFPLVWTNTCCSHPLYR.E | 2251.0686 |
| 87 | ENOYL-ACP REDUCTASE 1 family protein | *Populus trichocarpa* | 566147298 | 9.23/41.64 | 5.57/32.5 | 93 | 26.47 | 7 | R.VLAFEAGRK.N | 990.5468 |
|  |  |  |  |  |  |  |  |  | R.VNTISAGPLR.S | 1027.5596 |
|  |  |  |  |  |  |  |  |  | R.IIPGYGGGMSSAK.A | 1237.5773 |
|  |  |  |  |  |  |  |  |  | K.DLNIPTDKHQG.- | 1237.5773 |
|  |  |  |  |  |  |  |  |  | R.IIPGYGGGMSSAK.A + Oxidation (M) | 1253.5764 |
|  |  |  |  |  |  |  |  |  | K.VYPLDAVFDNLEDVPEDVK.A | 2176.9595 |
|  |  |  |  |  |  |  |  |  | K.AIGFIDTMIEYSLANAPLQK.E + Oxidation (M) | 2211.0308 |
| 90 | enoyl-ACP reductase | *Malus domestica* | 478435717 | 7.72/41.53 | 4.65/30.67 | 87 | 25.64 | 7 | R.VLAFEAGRK.H | 990.5468 |
|  |  |  |  |  |  |  |  |  | R.VNTISAGPLR.S | 1027.5596 |
|  |  |  |  |  |  |  |  |  | R.YLGSSNWTVK.E | 1154.5458 |
|  |  |  |  |  |  |  |  |  | R.IIPGYGGGMSSAK.A | 1237.5773 |
|  |  |  |  |  |  |  |  |  | R.IIPGYGGGMSSAK.A + Oxidation (M) | 1253.5764 |
|  |  |  |  |  |  |  |  |  | R.KLPDGSLMEITK.V | 1331.6727 |
|  |  |  |  |  |  |  |  |  | R.KLPDGSLMEITK.V + Oxidation (M) | 1347.6703 |
| **Unknown function** | | | | | | | | | | |
| 42 | hypothetical protein PRUPE_ppa012530mg | *Prunus persica* | 462414978 | 9.74/15.71 | 5.67/15.96 | 80 | 50 | 4 | R.SLLNEDEYGFR.K | 1342.6265 |
|  |  |  |  |  |  |  |  |  | R.LRGGNNVPTVPIK.S | 1364.6885 |
|  |  |  |  |  |  |  |  |  | R.RPIYYDEGLEK.T | 1382.6926 |
|  |  |  |  |  |  |  |  |  | K.ISQLNSAIDNVSSR.L | 1503.7733 |
| 45 | hypothetical protein PRUPE_ppa009297mg | *Prunus persica* | 462416990 | 5.36/32.32 | 5.76/34.36 | 231 | 58.62 | 12 | -.MAEARK.R | 705.3788 |
|  |  |  |  |  |  |  |  |  | K.KYPTFR.I | 811.4374 |
|  |  |  |  |  |  |  |  |  | R.GEVDWSK.L | 820.4561 |
|  |  |  |  |  |  |  |  |  | K.GIGLETVR.Q | 844.4874 |
|  |  |  |  |  |  |  |  |  | K.AALNAYTR.I | 879.4551 |
|  |  |  |  |  |  |  |  |  | K.GFTVVLTAR.D | 963.5519 |
|  |  |  |  |  |  |  |  |  | R.AVVESGAFGR.G | 992.5079 |
|  |  |  |  |  |  |  |  |  | K.GFPSSLSAYIVSK.A | 1355.7003 |
|  |  |  |  |  |  |  |  |  | K.VVFTDAENLTEER.L | 1522.7252 |
|  |  |  |  |  |  |  |  |  | R.TAEALIPLLQLSDSPR.I | 1723.9376 |
|  |  |  |  |  |  |  |  |  | K.TDINYNVGVLPVEEGAAR.V | 1916.9468 |
|  |  |  |  |  |  |  |  |  | K.LDILVNNAGIGGSTADPDAFR.A | 2116.0381 |
| 95 | hypothetical protein PRUPE_ppa017195mg | *Prunus persica* | 462408301 | 5.04/16.97 | 5.45/17.82 | 164 | 86.67 | 9 | -.MAVKEK.K | 705.3422 |
|  |  |  |  |  |  |  |  |  | K.IMPSIVR.R | 815.4242 |
|  |  |  |  |  |  |  |  |  | K.IMPSIVR.R + Oxidation (M) | 831.4176 |
|  |  |  |  |  |  |  |  |  | K.VLEGPALTLR.N | 1068.5698 |
|  |  |  |  |  |  |  |  |  | K.TVGVGVEALWR.A | 1186.5784 |
|  |  |  |  |  |  |  |  |  | K.IVELDDSEYR.F | 1238.5081 |
|  |  |  |  |  |  |  |  |  | K.IGEQETLVDMK.V | 1262.5400 |
|  |  |  |  |  |  |  |  |  | R.NFSALTTSFQLSK.I | 1443.6376 |
|  |  |  |  |  |  |  |  |  | K.EEANTGDIALQPAISYIQFLEK.Y | 2450.0459 |
| 71 | hypothetical protein PRUPE_ppa026808mg, partial | *Prunus persica* | 462406880 | 5.63/21.98 | 5.25/29.03 | 206 | 40 | 5 | K.VATAGQVFAVPR.G | 1215.6539 |
|  |  |  |  |  |  |  |  |  | K.TYLVDEDAINAMK.S | 1482.6893 |
|  |  |  |  |  |  |  |  |  | K.TYLVDEDAINAMK.S + Oxidation (M) | 1498.8286 |
|  |  |  |  |  |  |  |  |  | R.VDFGPGGINPPHSHPR.A | 1683.7976 |
|  |  |  |  |  |  |  |  |  | K.LPSEVTADDFVFDGLSK.Q | 1839.8685 |
| 67 | hypothetical protein PRUPE_ppa007643mg | *Prunus persica* | 462397640 | 7.57/39.57 | 5.4/30.87 | 163 | 61.11 | 17 | K.RDNPEYK.Y | 921.4898 |
|  |  |  |  |  |  |  |  |  | K.AFGMELLR.K | 936.4755 |
|  |  |  |  |  |  |  |  |  | K.AFGMELLR.K + Oxidation (M) | 952.4692 |
|  |  |  |  |  |  |  |  |  | R.RLLHVVYR.V | 1055.6276 |
|  |  |  |  |  |  |  |  |  | R.EPGPLPGINTK.I | 1122.5925 |
|  |  |  |  |  |  |  |  |  | K.FYTECLGMK.L | 1148.4875 |
|  |  |  |  |  |  |  |  |  | K.FYTECLGMK.L + Oxidation (M) | 1164.4863 |
|  |  |  |  |  |  |  |  |  | K.ITACLDPDGWK.S | 1275.5778 |
|  |  |  |  |  |  |  |  |  | K.SVFVDNVDFLK.E | 1282.6283 |
|  |  |  |  |  |  |  |  |  | K.YTIAMLGYGPEDK.N | 1457.6732 |
|  |  |  |  |  |  |  |  |  | K.YTIAMLGYGPEDK.N + Oxidation (M) | 1473.6935 |
|  |  |  |  |  |  |  |  |  | R.GPTPEPLCQVMLR.V | 1497.7231 |
|  |  |  |  |  |  |  |  |  | R.GPTPEPLCQVMLR.V + Oxidation (M) | 1513.7170 |
|  |  |  |  |  |  |  |  |  | K.GGNTVIAFVEDPDGYK.F | 1681.8290 |
|  |  |  |  |  |  |  |  |  | R.SITFYEKAFGMELLR.K + Oxidation (M) | 1820.8760 |
|  |  |  |  |  |  |  |  |  | K.YDIGTAFGHFGIAVDDVAK.T | 1995.9547 |
|  |  |  |  |  |  |  |  |  | K.GGNTVIAFVEDPDGYKFELLER.G | 2469.2000 |

**Spot no. corresponds to spots in Fig. 1, panel B; Homologous protein, Species, and Accession of the matched protein from the NCBI database; Theor pI/Mr, pI and molecular weight (kDa) annotated in the NCBI database; Exper pI/Mr, pI and molecular weight (kDa) found from the gel and analyzed using Image Master and Melanie software; Score, the score obtained from the NCBI database for each match; C(%), percentage of the conserved region of the identified peptide sequences in the matched protein; PN, number of matched peptides; Peptide sequence, sequences of the matched peptides; Observed m/z, the mass/charge ratio.**
