# Supplementary material for: Physiological and proteomic analyses of the drought stress response in Amygdalus Mira (Koehne) Yü et Lu roots
Source: BMC Plant Biol. 2017 Feb 27;17:53. doi: 10.1186/s12870-017-1000-z (PMC5327565; doi:10.1186/s12870-017-1000-z)
Supplement: Additional file 2: Table S2. — The primer sequences for real time PCR. (DOC 41 kb) [file 12870_2017_1000_MOESM2_ESM.doc]

**Supplemental Table S1: The primer sequences for real time PCR. Spot no. corresponding to spots in Fig. 1, panel B.**

| Spot No. | Forward (5'-3') | Reverse (5'-3') | Annealing temperature (℃) | Cycle  number |
| --- | --- | --- | --- | --- |
| ACTIN | ATTGTGAGCAACTGGGATG | CTGACACCATCTCCAGAGTC | 53 | 32 |
| Spot.42 | GCTGGCTTATTTTTGCATTC | TCTTAGGTGGTGCTGTCTTTG | 53 | 40 |
| Spot.61 | GTGACCGGTGTTGAGATGTT | TTCCAGTGATATCTGCAGTCC | 56 | 40 |
| Spot.76 | TATGGTTTGGACAAGAAGGG | GCTCTGTTGTCCTTGCTGAT | 55 | 40 |
| Spot.11 | GATTCGTGAAGAACGTGGT | ACATCAAAATAACCACTTCCA | 54 | 40 |
| Spot.15 | GGCAGCGTTTGGTCTCAG | CCCTTCTTTCCTCGAATGAC | 56 | 40 |
| Spot.51 | GTGTTGGAACCATCAAGAAGA | TCCTCTTCCTTGATCTCAACA | 56 | 40 |
| Spot.75 | AGCAGGAGGAGAAGAACGAC | AATGGCCTTGACCTGTGG | 56 | 40 |
| Spot.70 | GACGAATGTATTCTGGTGGAT | CCTTTGAGCAGCATTTCTTAC | 56 | 40 |
| Spot.90 | AGCATTGATATCCTTGTCCAC | GTCACTCTCTAGAGCAGCCTT | 56 | 40 |
| Spot.45 | GGTGCTAAAAGAACAGCTGAA | ATATCTGTTTTGACAAAGCCAG | 54 | 40 |
| Spot.59 | AGGAGTTCATGATTCTTCCTG | CCATAAAACTCAGATGCAGCA | 56 | 40 |
