# Supplementary material for: Physiological and proteomic analyses of the drought stress response in Amygdalus Mira (Koehne) Yü et Lu roots
Source: BMC Plant Biol. 2017 Feb 27;17:53. doi: 10.1186/s12870-017-1000-z (PMC5327565; doi:10.1186/s12870-017-1000-z)
Supplement: Additional file 3: Table S3. — Proteins differentially expressed in Amygdalus mira (Koehne) Yü et Lu during drought and recovery. (DOC 229 kb) [file 12870_2017_1000_MOESM3_ESM.doc]

**Table. 1 Proteins differentially expressed in *Amygdalus mira (Koehne) Yü et Lu* during drought and recovery.**

| **Spot no.** | **Homologous protein** | **D4** | **D8** | **D12** | **D16** | **D20** |
| --- | --- | --- | --- | --- | --- | --- |
| **Cytoskeleton dynamics** | |  |  |  |  |  |
| 40 | profilin | 0.998±0.012 | 0.696±0.019 | 0.574±0.022 | 0.430±0.008 | 0.775±0.012 |
| 84 | actin 1 | 0.993±0.011 | 0.565±0.013 | 0.243±0.007 | 0.176±0.008 | 1.010±0.013 |
| 85 | ACT1 | 1.000±0.011 | 0.823±0.015 | 0.923±0.011 | 0.453±0.010 | 1.354±0.012 |
| **Carbohydrate and nitrogen metabolism** | |  |  |  |  |  |
| 20 | Succinyl-CoA ligase beta-chain family protein | 0.951±0.105 | 0.754±0.083 | 0.577±0.101 | 0.493±0.005 | 2.022±0.092 |
| 26 | beta-hexosaminidase 2 | 0.962±0.093 | 2.282±0.188 | 1.744±0.075 | 2.916±0.229 | 2.156±0.130 |
| 38 | alcohol dehydrogenase | 1.000±0.087 | 0.821±0.101 | 0.419±0.111 | 0.313±0.072 | 0.227±0.036 |
| 59 | enolase | 0.993±0.020 | 1.286±0.017 | 2.640±0.217 | 3.850±0.183 | 0.911±0.093 |
| 91 | Glyceraldehyde-3-phosphate dehydrogenase | 0.992±0.013 | 0.670±0.033 | 0.553±0.015 | 0.410±0.009 | 0.944±0.013 |
| 92 | cytosolic aldolase | 0.994±0.009 | 0.540±0.015 | 0.358±0.007 | 0.265±0.009 | 0.966±0.010 |
| **Energy metabolism** | |  |  |  |  |  |
| 1 | ATP synthase beta subunit | 0.907±0.107 | 2.492±0.161 | 2.816±0.155 | 5.960±0.237 | 0.781±0.109 |
| 13 | cytochrome P450 | 0.738±0.099 | 1.947±0.041 | 3.823±0.279 | 6.835±0.288 | 1.081±0.093 |
| 46 | cytochrome c oxidase subunit | 1.000±0.037 | 0.482±0.047 | 0.540±0.027 | 0.068±0.007 | 2.758±0.073 |
| 48 | PREDICTED: uncharacterized mitochondrial protein AtMg00820-like | 1.030±0.023 | 0.508±0.017 | 0.342±0.011 | 0.166±0.008 | 1.748±0.021 |
| 56 | ATP synthase beta subunit | 1.084±0.017 | 1.749±0.031 | 1.675±0.025 | 2.344±0.040 | 0.814±0.016 |
| 47 | Biotin carboxylase | 0.981±0.012 | 0.555±0.009 | 0.383±0.009 | 0.079±0.004 | 0.879±0.013 |
| 22 | Cobalt import ATP-binding protein cbiO, putative | 0.983±0.020 | 0.533±0.018 | 0.261±0.014 | 0.067±0.004 | 2.381±0.049 |
| **Transcription and translation** | |  |  |  |  |  |
| 3 | ORF; able to induce HR-like lesions | 0.943±0.009 | 1.260±0.016 | 1.421±0.020 | 4.771±0.082 | 0.398±0.009 |
| 8 | WRKY transcription factor 19 | 1.497±0.109 | 1.792±0.021 | 4.745±0.158 | 4.375±0.217 | 0.587±0.041 |
| 9 | WRKY transcription factor 19 | 1.073±0.033 | 1.843±0.021 | 1.868±0.017 | 3.757±0.201 | 0.069±0.011 |
| 19 | dead box ATP-dependent RNA helicase, putative | 1.055±0.014 | 0.700±0.013 | 0.403±0.009 | 0.105±0.005 | 2.238±0.022 |
| 28 | PREDICTED: ethylene-responsive transcription factor 1A-like | 0.961±0.018 | 2.827±0.041 | 6.544±0.039 | 11.415±0.309 | 2.192±0.117 |
| 29 | histone-like protein | 0.989±0.009 | 10.035±0.291 | 10.331±0.317 | 16.169±0.588 | 2.832±0.017 |
| 49 | retrotransposon protein, putative, Ty3-gypsy subclass | 1.002±0.011 | 0.530±0.011 | 0.366±0.010 | 0.146±0.006 | 0.716±0.010 |
| 54 | ribosomal protein S18 | 1.018±0.009 | 3.301±0.047 | 4.042±0.039 | 31.828±1.831 | 0.899±0.024 |
| 57 | Mitochondrial HSO70 2 isoform 2 | 0.995±0.008 | 1.794±0.012 | 2.352±0.033 | 5.264±0.205 | 1.213±0.023 |
| 58 | PREDICTED: ribonuclease R-like | 0.974±0.103 | 1.470±0.051 | 2.112±0.028 | 3.165±0.233 | 1.343±0.020 |
| 61 | Elongation factor Tu family protein | 0.983±0.111 | 1.704±0.073 | 4.673±0.195 | 4.532±0.201 | 1.703±0.039 |
| 64 | putative XH domain family protein | 0.984±0.039 | 1.776±0.017 | 3.026±0.177 | 4.249±0.195 | 1.595±0.029 |
| 66 | signal recognition particle 54 kDa subunit | 1.001±0.011 | 1.550±0.019 | 3.625±0.137 | 8.525±0.316 | 0.874±0.022 |
| 69 | Transcription-repair-coupling factor | 1.005±0.038 | 1.797±0.028 | 3.930±0.148 | 10.430±0.424 | 1.407±0.025 |
| 23 | HAT family dimerization domain containing protein | 0.956±0.031 | 0.741±0.012 | 0.537±0.015 | 0.251±0.009 | 2.231±0.047 |
| 76 | ER-binding protein | 1.002±0.021 | 0.784±0.014 | 0.370±0.010 | 0.293±0.007 | 1.309±0.017 |
| 79 | RNA-binding KH domain-containing protein | 1.024±0.013 | 0.678±0.012 | 0.735±0.017 | 0.444±0.008 | 0.378±0.008 |
| 89 | NAC transcription factor 1 | 1.000±0.007 | 0.653±0.017 | 0.513±0.009 | 0.386±0.010 | 0.595±0.034 |
| **Transport** |  |  |  |  |  |  |
| 4 | clathrin assembly protein | 1.023±0.014 | 1.404±0.033 | 3.315±0.116 | 2.856±0.109 | 0.432±0.008 |
| 5 | UDP-L-arabinose mutase-like protein | 1.275±0.062 | 2.296±0.123 | 5.166±0.152 | 14.498±1.046 | 0.182±0.004 |
| 17 | DYNAMIN-LIKE protein 5 | 0.966±0.028 | 0.778±0.016 | 0.358±0.010 | 0.181±0.010 | 2.346±0.093 |
| 83 | alpha-tubulin 4 | 0.993±0.013 | 0.594±0.010 | 0.163±0.006 | 0.143±0.007 | 0.902±0.010 |
| **Inducer** |  |  |  |  |  |  |
| 10 | induced stolon tip protein PJ-1 | 0.914±0.017 | 1.389±0.011 | 1.624±0.027 | 2.331±0.021 | 0.347±0.014 |
| 35 | induced stolon tip protein PJ-1 | 0.932±0.023 | 0.825±0.017 | 3.789±0.128 | 0.401±0.017 | 0.077±0.004 |
| 37 | induced stolon tip protein PJ-1 | 1.028±0.131 | 0.600±0.067 | 0.473±0.023 | 0.217±0.010 | 0.387±0.017 |
| **Stress and defense** | |  |  |  |  |  |
| 2 | superoxide dismutase [Cu-Zn] 1 | 0.935±0.027 | 0.594±0.019 | 0.349±0.008 | 0.284±0.011 | 0.771±0.023 |
| 6 | PREDICTED: arginase 1, mitochondrial-like isoform X3 | 0.840±0.019 | 2.362±0.151 | 3.578±0.269 | 4.665±0.242 | 1.340±0.031 |
| 11 | PREDICTED: 1,2-dihydroxy-3-keto-5-methylthiopentene dioxygenase 3-like isoform X1 | 0.702±0.014 | 4.921±0.178 | 4.089±0.262 | 7.564±0.372 | 0.517±0.013 |
| 12 | NBS-LRR-like protein, partial | 1.089±0.018 | 2.901±0.133 | 8.820±0.199 | 12.750±0.303 | 0.074±0.015 |
| 14 | metallothionin 3 | 0.976±0.022 | 1.971±0.017 | 2.665±0.125 | 3.019±0.148 | 0.282±0.011 |
| 15 | putative Pru du 4.1 allergen, partial | 0.914±0.019 | 9.683±0.307 | 10.000±0.335 | 22.754±0.518 | 0.237±0.011 |
| 18 | nectarin 5 | 0.962±0.022 | 0.511±0.015 | 0.377±0.013 | 0.211±0.008 | 2.112±0.021 |
| 24 | Glycin-rich RNA binding protein | 0.978±0.013 | 0.604±0.017 | 0.308±0.021 | 0.018±0.004 | 4.214±0.138 |
| 27 | germin-like protein subfamily 1 member 1 precursor | 1.017±0.013 | 1.302±0.104 | 1.839±0.022 | 2.511±0.028 | 13.252±0.273 |
| 31 | metallothionin 3 | 1.067±0.021 | 2.924±0.101 | 3.852±0.174 | 6.419±0.266 | 4.859±0.132 |
| 34 | dihydrolipoamide dehydrogenase | 0.998±0.013 | 4.434±0.094 | 5.179±0.187 | 6.061±0.219 | 0.386±0.018 |
| 36 | retrotransposon protein, putative, Ty3-gypsy subclass | 0.991±0.011 | 0.814±0.013 | 0.547±0.024 | 0.042±0.006 | 0.059±0.006 |
| 39 | 1-aminocyclopropane-1-carboxylate oxidase | 0.982±0.019 | 0.773±0.023 | 0.045±0.003 | 0.194±0.017 | 0.296±0.010 |
| 44 | Peroxisomal membrane protein PMP22 | 1.000±0.008 | 0.502±0.018 | 0.285±0.012 | 0.071±0.004 | 0.918±0.028 |
| 50 | pathogenesis related protein PR10 | 0.989±0.015 | 0.660±0.017 | 0.408±0.011 | 0.249±0.021 | 1.000±0.008 |
| 51 | major cherry allergen Pru av 1.0201 | 1.000±0.012 | 0.684±0.020 | 0.475±0.017 | 0.208±0.007 | 0.792±0.014 |
| 53 | thioredoxin h-type | 1.003±0.011 | 12.537±1.572 | 41.818±2.773 | 65.926±4.191 | 0.398±0.016 |
| 55 | metallothionin 3 | 0.993±0.017 | 1.771±0.031 | 2.280±0.153 | 4.329±0.244 | 1.041±0.018 |
| 63 | ascorbate peroxidase | 0.921±0.021 | 1.504±0.019 | 1.962±0.017 | 2.550±0.026 | 0.699±0.017 |
| 68 | thaumatin-like protein 2 | 0.998±0.014 | 1.132±0.031 | 12.390±0.628 | 13.187±0.599 | 0.818±0.014 |
| 60 | resistance protein | 1.013±0.009 | 1.696±0.023 | 1.985±0.017 | 2.219±0.025 | 0.430±0.010 |
| 77 | PREDICTED: brefeldin A resistance protein-like | 0.977±0.017 | 0.713±0.014 | 0.305±0.008 | 0.304±0.010 | 0.772±0.016 |
| 73 | Catalase isozyme 2 | 0.999±0.012 | 6.370±0.197 | 16.126±0.793 | 19.762±0.688 | 0.654±0.017 |
| 88 | Catalase isozyme 2 | 0.999±0.007 | 5.727±0.327 | 0.236±0.011 | 0.215±0.004 | 0.698±0.014 |
| 86 | senescence-associated protein 6 | 1.025±0.013 | 0.674±0.017 | 0.575±0.008 | 0.419±0.010 | 4.737±0.228 |
| **Molecular chaperones** | |  |  |  |  |  |
| 30 | Chaperone protein dnaJ | 0.966±0.018 | 5.543±0.173 | 1.025±0.014 | 18.174±0.438 | 3.525±0.383 |
| 32 | cytosolic class I small heat shock protein type 1 | 0.975±0.012 | 4.952±0.201 | 0.355±0.012 | 8.684±0.315 | 1.532±0.026 |
| 75 | small heat shock protein | 1.038±0.015 | 1.986±0.028 | 3.087±0.121 | 4.131±0.173 | 1.963±0.024 |
| 78 | Stromal 70 kDa heat shock-related family protein | 0.993±0.009 | 0.797±0.012 | 0.444±0.012 | 0.363±0.010 | 1.503±0.021 |
| 80 | endoplasmin, putative | 1.001±0.012 | 0.893±0.011 | 0.444±0.014 | 0.377±0.007 | 0.775±0.011 |
| 81 | heat shock cognate protein 70 | 0.994±0.017 | 0.459±0.010 | 0.264±0.007 | 0.220±0.007 | 0.390±0.008 |
| 82 | Stromal 70 kDa heat shock-related family protein | 0.993±0.013 | 0.171±0.008 | 0.072±0.002 | 0.050±0.002 | 0.759±0.012 |
| 94 | copper chaperone | 0.997±0.012 | 0.543±0.009 | 0.493±0.014 | 0.324±0.006 | 4.695±0.155 |
| 96 | Luminal-binding protein 5 | 0.978±0.017 | 0.675±0.013 | 0.356±0.011 | 0.313±0.013 | 0.927±0.027 |
| **Protein degradation** | |  |  |  |  |  |
| 16 | PREDICTED: putative DNA repair protein RAD23-3-like | 0.956±0.023 | 0.519±0.017 | 0.106±0.004 | 0.037±0.002 | 9.954±0.216 |
| 25 | RAD23 protein | 0.782±0.027 | 30.130±0.994 | 67.610±2.384 | 63.627±1.959 | 0.780±0.018 |
| 72 | proteasome subunit alpha type-5 | 0.999±0.011 | 1.862±0.026 | 3.443±0.108 | 4.830±0.147 | 0.976±0.015 |
| **Signal transduction** | |  |  |  |  |  |
| 21 | Glycogen synthase kinase-3 beta, putative | 0.944±0.017 | 0.764±0.020 | 0.331±0.014 | 0.271±0.012 | 2.692±0.087 |
| 33 | Calcium-binding EF-hand family protein | 0.999±0.007 | 3.203±0.063 | 4.152±0.142 | 4.242±0.137 | 2.098±0.073 |
| 41 | Transducin/WD40 repeat-like superfamily protein, putative | 0.997±0.010 | 0.653±0.018 | 0.304±0.013 | 0.0421±0.001 | 0.672±0.016 |
| 43 | ethylene receptor-like protein precursor | 0.994±0.020 | 0.850±0.017 | 0.584±0.012 | 0.292±0.005 | 1.030±0.012 |
| 62 | tyrosine-specific protein phosphatase-like protein | 0.988±0.011 | 1.761±0.024 | 3.069±0.083 | 3.606±0.111 | 1.268±0.008 |
| 74 | calcineurin B-like | 1.005±0.006 | 1.878±0.016 | 1.417±0.013 | 2.162±0.019 | 1.571±0.013 |
| 93 | TCTP protein | 0.989±0.013 | 0.718±0.027 | 0.519±0.009 | 0.454±0.011 | 0.667±0.008 |
| **Other Materials Metabolism** | |  |  |  |  |  |
| 7 | O-acetylserine(thiol)-lyase | 0.842±0.022 | 2.840±0.032 | 2.583±0.028 | 4.129±0.162 | 0.287±0.004 |
| 65 | caleosin CLO1-6 | 1.017±0.010 | 1.497±0.018 | 1.877±0.015 | 2.248±0.011 | 1.193±0.010 |
| 70 | isopentenyl diphosphate isomerase | 1.034±0.007 | 2.977±0.026 | 4.019±0.038 | 5.622±0.123 | 1.744±0.008 |
| 87 | ENOYL-ACP REDUCTASE 1 family protein | 1.021±0.016 | 0.774±0.009 | 0.609±0.013 | 0.389±0.011 | 0.837±0.011 |
| 90 | enoyl-ACP reductase | 1.008±0.013 | 5.058±0.171 | 0.381±0.005 | 0.213±0.005 | 0.893±0.012 |
| **Unknown function** | |  |  |  |  |  |
| 42 | hypothetical protein PRUPE_ppa012530mg | 0.971±0.021 | 0.495±0.016 | 0.183±0.002 | 0.027±0.001 | 0.725±0.010 |
| 45 | hypothetical protein PRUPE_ppa009297mg | 1.004±0.007 | 0.677±0.018 | 0.567±0.013 | 0.046±0.003 | 0.989±0.014 |
| 95 | hypothetical protein PRUPE_ppa017195mg | 0.997±0.010 | 0.844±0.009 | 0.533±0.016 | 0.397±0.018 | 0.705±0.022 |
| 71 | hypothetical protein PRUPE_ppa026808mg, partial | 1.002±0.014 | 1.418±0.020 | 1.526±0.017 | 2.930±0.103 | 0.846±0.015 |
| 67 | hypothetical protein PRUPE_ppa007643mg | 0.995±0.014 | 1.463±0.009 | 1.789±0.010 | 3.205±0.085 | 1.214±0.013 |

**Spot no. corresponding to spots in Fig. 1, panel B; Homologous protein, matched protein from the NCBI database; D4, D8, D12, D16, D20, the average fold times in spot intensities on day 4, 8, 12, 16 and 20 respectively after starting the stress compared with control groups.**
