# Supplementary material for: Physiological and proteomic analyses of the drought stress response in Amygdalus Mira (Koehne) Yü et Lu roots
Source: BMC Plant Biol. 2017 Feb 27;17:53. doi: 10.1186/s12870-017-1000-z (PMC5327565; doi:10.1186/s12870-017-1000-z)
Supplement: Additional file 4: Figure S4. — Semi-quantitative PCR analysis of eleven genes and ACTIN of Amygdalus mira (Koehne) Yü et Lu roots during drought stress and recovery period, respectively. (DOC 201 kb) [file 12870_2017_1000_MOESM4_ESM.doc]

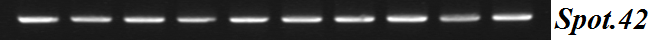


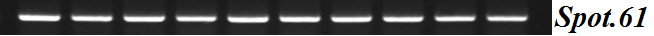


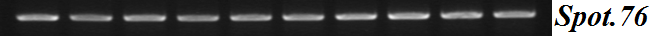


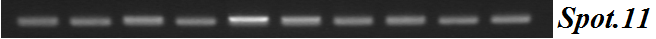


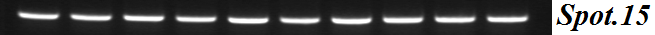


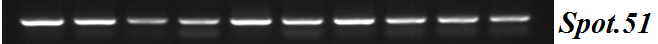


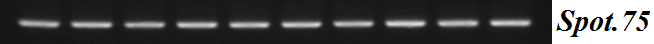


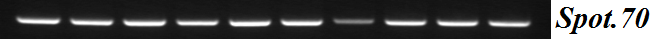


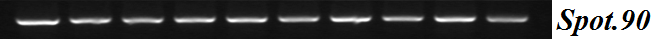


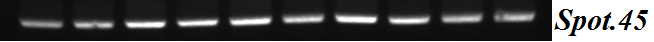


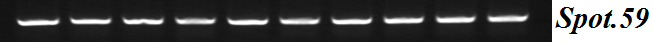


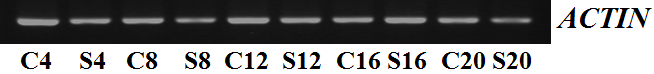


**Supplemental Fig. 1. Semi-quantitative PCR analysis of eleven genes including *Spot.42, Spot.61, Spot.76, Spot.11, Spot.15, Spot.51, Spot.75, Spot.70, Spot.90, Spot.45, Spot.59* and *ACTIN* of *Amygdalus mira (Koehne) Yü et Lu* roots during drought stress and recovery period, respectively. Spot no. corresponding to spots in Fig. 1, panel B.** **C4, control at day 4; S4, treatment at day 4; C8, control at day 8; S8, treatment at day 8; C12, control at day 12; S12, treatment at day 12; C16, control at day 16; S16, treatment at day 16; C20, control at day 20; S20, treatment at day 20;**
